# Consistent traffic noise impacts few fitness-related traits in a field cricket

**DOI:** 10.1186/s12862-023-02190-2

**Published:** 2023-12-20

**Authors:** Gabrielle T. Welsh, Sophia C. Anner, Mary L. Westwood, Victoria Rockwell, Hannah O’Toole, Megan Holiday, Robin M. Tinghitella

**Affiliations:** 1 Department of Biological Sciences, University of Denver, Denver, CO USA; 2 Department of Biology, University of Louisville, Louisville, KY USA; 3 Department of Physics, University of Oxford, Oxford, UK

**Keywords:** Anthropogenic noise, Life history, Reproduction, Immunity, *Orthoptera*

## Abstract

**Background:**

Anthropogenic habitat change is occurring rapidly, and organisms can respond through within-generation responses that improve the match between their phenotype and the novel conditions they encounter. But, plastic responses can be adaptive or maladaptive and are most likely to be adaptive only when contemporary conditions reasonably mimic something experienced historically to which a response has already evolved. Noise pollution is a ubiquitous anthropogenic stressor that accompanies expanding urbanization. We tested whether the amplitude of traffic noise influences a suite of fitness-related traits (e.g. survival, life history, reproductive investment, immunity) and whether that depends on the life stage at which the noise is experienced (juvenile or adult). Our treatments mimic the conditions experienced by animals living in urban roadside environments with variable vehicle types, but continuous movement of traffic. We used the Pacific field cricket, an acoustically communicating insect that was previously shown to experience some negative behavioral and life history responses to very loud, variable traffic noise, as a model system.

**Results:**

After exposing crickets to one of four traffic noise levels (silence, 50dBA, 60dBA, and 70dBA which are commonly experienced in their natural environment) during development, at adulthood, or both, we measured a comprehensive suite of fifteen fitness-related traits. We found that survival to adulthood was lower under some noise treatments than under silence, and that the number of live offspring hatched depended on the interaction between a female’s juvenile and adult exposure to traffic noise. Both of these suggest that our noise treatments were indeed a stressor. However, we found no evidence of negative or positive fitness effects of noise on the other thirteen measured traits.

**Conclusions:**

Our results suggest that, in contrast to previous work with loud, variable traffic noise, when noise exposure is relatively constant, plasticity may be sufficient to buffer many negative fitness effects and/or animals may be able to habituate to these conditions, regardless of amplitude. Our work highlights the importance of understanding how the particular characteristics of noise experienced by animals influence their biological responses and provides insight into how commensal animals thrive in human-dominated habitats.

**Supplementary Information:**

The online version contains supplementary material available at 10.1186/s12862-023-02190-2.

## Background

Urbanization is increasing dramatically [1] and is expected to continue expanding (discussed in [2]). The pace and extent of urbanization makes understanding how anthropogenic influences affect human-adjacent animal communities an urgent area of investigation [3]. When faced with human-induced environmental change, animals can disperse to new environments, adapt to local conditions, or respond through within- or between-generation phenotypic plasticity [4, 5]. Dispersal is often costly or impossible (depending on locomotor abilities, available escape routes, etc.), and while adaptive evolution can sometimes keep pace with anthropogenic change, it often operates too slowly to be beneficial [5]. Phenotypic plasticity (the within-generation process by which one individual or genotype can produce multiple phenotypes [6]) allows organisms to adjust to an environment without changes to their genetic make-up. It can also help ameliorate negative impacts of urbanization on organisms’ fitness by acting as a ‘buffer’ to varied environmental conditions and/or allowing time for evolutionary change to occur [7, 8]. For instance, organisms that migrate between rural and urban areas are more likely to acclimate (adjust traits to environmental field conditions) to urban environments via phenotypic plasticity than adapt to them [9, 10]. However, many phenotypic modifications to anthropogenically impacted environments are imperfect and can even be costly or occur in seemingly maladaptive directions [11], leaving open the question of whether responses to urbanization facilitate or hinder life in urban settings.

Organisms adjust phenotypes in response to environments encountered at all life stages. Phenotypic change stemming from experience during development can influence adult traits and may be particularly important for persistence under novel environmental conditions if early experience prepares organisms to live successfully in the environments they will experience as adults [12, 13]. Phenotypic adjustment to environments experienced as adults may also be important and is often reversible, which is valuable when environments change often, rapidly, or are unpredictable across an individual’s lifespan [14]. But what are the relative roles of exposure to urban stressors during development and at adulthood? Do developmental and adult experiences interact with one another to structure fitness-related traits? Do they impact different traits? Moreover, plasticity is itself an evolved trait and can be costly or constrained [15, 16], so within-generation responses may only be adaptive when the current stressor reasonably mimics something that has been experienced historically, and to which an adaptive response has already evolved [17, 18]. Thus, the answers to these questions may depend on the type or degree of stressor experienced.

One major consequence of expanding urbanization is noise pollution. Currently, more than 83% of land in the continental US is exposed to vehicular noise [19], and 88% of people are exposed to anthropogenic noise louder than 55dBA [20], which is roughly equivalent to the sound of constant rainfall. Noise pollution impacts numerous behavioral, physiological, and fitness-related survival and reproductive traits of animals [21–24]. For instance, anthropogenic noise leads to shifts in critical vocal communication in various bird [25, 26], insect [27, 28], and anuran species [29, 30], increases stress hormone levels in birds and fish [31, 32], and reduces clutch sizes of female great tits [33]. Noise pollution has even been associated with declines in arthropod abundance, which could have drastic impacts on entire ecological communities of interacting organisms [34, 35]. While most studies have looked only at the impacts of very loud noise (usually 70-80dBA) on animals [2], we seek to understand the impacts of the range of noise levels commonly experienced by animals living in suburban and urban environments (see, for example [28, 36, 37]). Grasshoppers (*Chorthippus biguttulus)* living in northwestern Germany, for instance, experience mean maximum background noise of 37.5-54.2dBA away from roadsides, but 78.9-87.0dBA very near roadsides [27], so clearly the acoustic environment varies substantially in nature. An experimental approach that exposes animals to a range of noise amplitudes will allow us to address whether the characteristics of noise encountered affect within-generation responses of fitness related traits. Further, until recently, most noise pollution research has been conducted in vertebrates—a 2016 review found that only 4% of work on the effects of anthropogenic noise on terrestrial species addressed effects on invertebrates [2]. Yet, invertebrates comprise over 90% of all animals [38], are immensely diverse, and play important roles as food resources, predators, parasites, pollinators, and pests or pest control [39]; all of this underscores the importance of understanding their responses to anthropogenic disturbances like noise [40–42]. Acoustically communicating insects may be particularly impacted by noise pollution because they rely on sound to communicate intraspecifically [43]. One such insect group, field crickets, produces airborne acoustic songs in intraspecific communication [44, 45]. Many field crickets’ songs overlap in frequency (pitch) with traffic noise [46, 47]. Crickets’ hearing abilities also overlap with the frequency range of traffic noise (e.g. [48, 49] and reviewed in [43]), which means noise could also interfere with non-sexual aspects of life like detecting predators.

The Pacific field cricket (*Teleogryllus oceanicus*) lives in varied habitats, ranging from undisturbed rural areas on isolated Pacific islands to agricultural areas and even vacant lots in major cities (discussed in [46]). As a result, it experiences a variety of noise environments in nature. Previous work in this study system revealed some negative effects of loud (~ 70dBA) traffic noise experienced during development, or chronically (24 h a day) throughout life, on life history and behavior. Pacific field crickets reared in loud traffic noise throughout their lives took 23% longer to reach adulthood and had 13% shorter adult lifespans [50] than those reared in the absence of traffic noise. Further, females exposed to that same traffic noise only during development took 80% longer to reach singing males when searching for mates as adults, suggesting that developmental exposure to noise hindered, rather than prepared, females to search for mates under noise [50]. Similar behavioral costs of exposure to noise have been observed in other field crickets. In *Gryllus bimaculatus*, crickets that experienced traffic noise during phonotaxis and mate choice trials were less likely to approach male calls and had a reduced preference for high-quality male songs in comparison to those that experienced ambient noise [51, 52]. Notably, the average amplitude of the traffic noise broadcast in previous studies with *T. oceanicus* was at the upper end of what appears to be experienced in urban environments [36], and the traffic track played back contained frequent changes in amplitude [46]. It best mimicked a roadside environment with discontinuous movement of traffic with a high number of vehicle accelerations and decelerations. Insects living in many urban habitats, however, are more likely to encounter predictable, relatively constant traffic or other urban noise (e.g. those living close to highways on which vehicles move at steady high speeds), and animals further from point sources of consistent noise experience that predictable, relatively constant noise at lower amplitudes. Like other Orthopterans (e.g. [27, 28, 36]), *T. oceanicus* does live very near roadsides, and in some cases those roadside environments likely expose them to the consistent traffic noise characteristic of high fluidity of traffic (personal observation, RT).

**Fig. 1:**
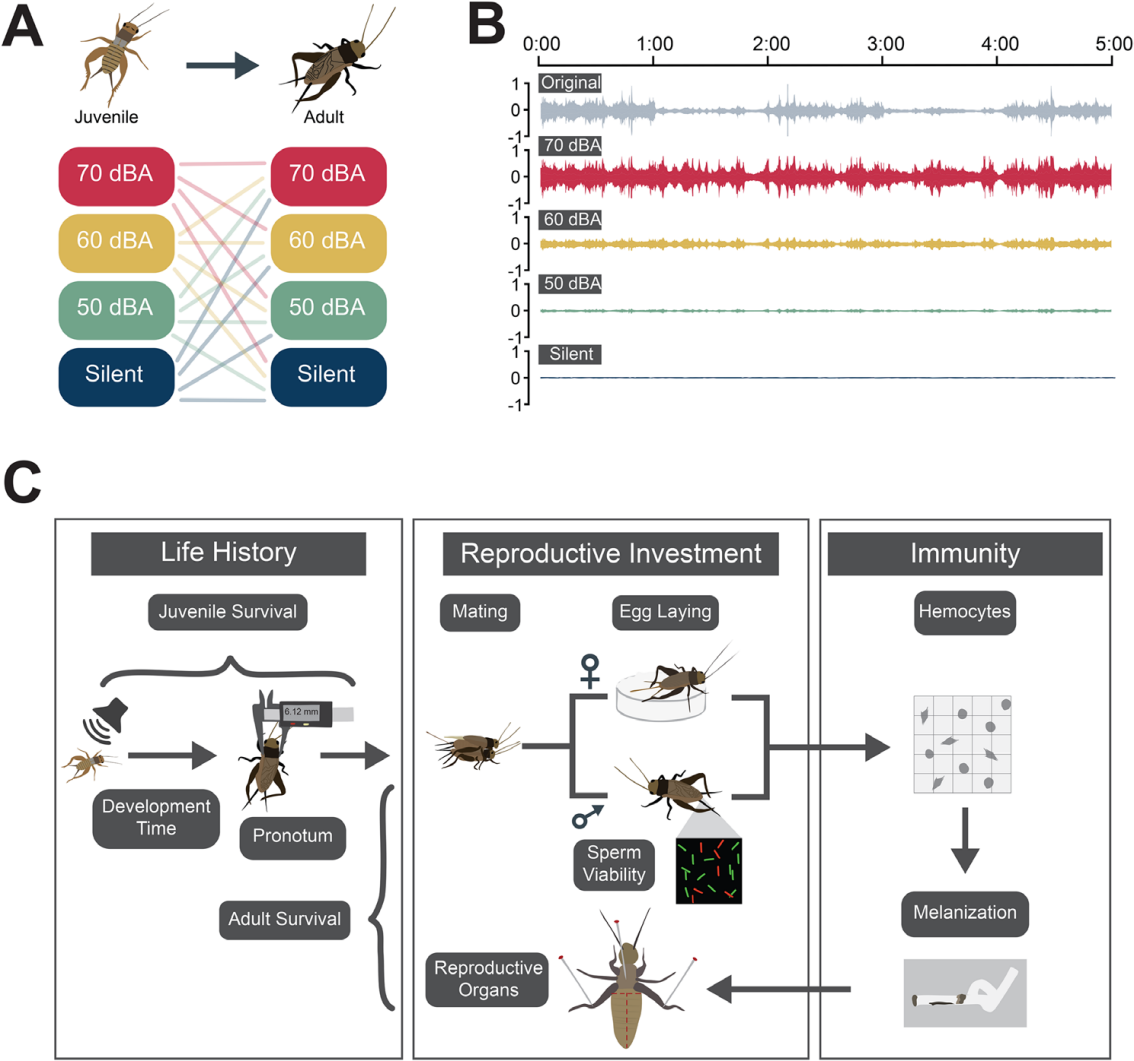
Summary of experimental design and workflow. (**A**) We exposed crickets to no traffic noise or one of the three amplitudes of traffic noise as juveniles and then re-assigned them to noise treatments as adults. (**B**) To broadcast traffic noise at different amplitudes across treatments, we first leveled our traffic noise recordings to achieve more consistency in amplitude during playback. We then created three traffic noise tracks from this leveled recording, corresponding to 50dBA, 60dBA, and 70dBA at one meter from the source of sound, respectively. The tracks still contain substantial variation in spectral content over time, reflecting a diverse vehicular makeup (see Supplementary Fig. 1). (**C**) At adulthood, we assayed 15 fitness-related traits

We used the Pacific field cricket system to test whether and how the severity (here, amplitude of noise) and timing (e.g. during development versus adulthood) of anthropogenic noise impacts fitness-related traits and focused on the effects of predictable, relatively constant traffic noise. We exposed Pacific field crickets to noise at amplitudes commonly encountered in urban areas during development, adulthood, both, or neither (Fig. 1a). We then assessed the impacts of those noise exposures on a comprehensive suite of life-history, reproductive, and immune-related traits, some of which have not yet been investigated in the insect noise pollution literature and many of which are known to be plastic (e.g. sperm viability, immunity, and reproductive investment [53, 54]). Finding a significant effect of developmental or adult exposure would indicate that exposure to anthropogenic noise impacts fitness-related traits. As is the case with other anthropogenic stressors, plastic responses may be in an adaptive direction (increasing fitness above the no noise treatment) or a non-adaptive direction (reducing fitness relative to the no noise treatment) [11]. Our design also allows us to determine whether plastic responses to noise depend on the level of the stressor experienced (amplitude of noise), whether plastic responses are stage dependent (a response to developmental and/or adult exposure), and whether experience with noise at different stages of life interact with one another to determine fitness-related adult traits. Alternatively, we may find no significant effects of noise on some fitness-related traits. Interestingly, though, finding no effects of noise may actually reflect effective plastic responses that buffer against otherwise negative impacts of noise on fitness; such a result would add valuable information to our understanding of how commensal organisms persist in suburban and urban environments despite human-generated stressors in these places.

## Methods

### General experimental design

We used a fully factorial design with four possible noise levels experienced during development and/or at adulthood (Fig. 1a). We assigned juvenile crickets to one of four acoustic environments: a no traffic noise treatment or one of three chronic traffic noise treatments (50dBA, 60dBA, or 70dBA). These noise levels span those most commonly experienced in urbanizing environments; the Environmental Protection Agency defines 55dBA as an acceptable outdoor noise level [55], and 70dBA is the most common amplitude investigated in studies of noise pollution including previous studies in *T. oceanicus* [2]. At adulthood, we randomly reassigned the crickets to a noise treatment (mimicking the potential for the animals to disperse by flight at adulthood, which they cannot do as juveniles) and then measured a suite of fitness-related traits, which we categorized as related to basic life-history (survival, development time and adult size), reproductive investment (mating success, sperm viability, number of eggs hatched after 1 week of laying, and male and female investment in reproductive organs), and immunity (hemocyte counts and melanization of a foreign body).

### Rearing and traffic noise exposure

To expose crickets to different noise levels, we reared the animals inside of replicate, acoustically isolated Percival incubators (model I36VLC). Because the incubators themselves generate white noise when on [46], we left the power off, but retained a 12:12 light:dark schedule in each using clamp lights outfitted with 60 W LED bulbs attached to mechanical timers. The lighting also maintained incubator temperatures within those naturally experienced by the animals (21 to 23 °C), and we maintained 60–65% humidity by placing a 1.89 L bin of water in each incubator. We measured temperature and humidity every two weeks.

Each acoustically isolated incubator housed a Bluetooth EcoXBT speaker that either played nothing (leaving background ambient noise of 37-38dBA only; hereafter “silent”) or an uncompressed. wav traffic noise track standardized to 50dBA, 60dBA, or 70dBA (when measured at 1 m away). We created the traffic tracks following [46, 50] and used the same traffic noise recordings as those studies. In brief, we first spliced together ten randomly chosen 30 s clips of traffic noise originally recorded for [46] to create a five-minute track. To investigate how noise levels impact plastic responses to noise, we next leveled the sound from each recording clip so that the average amplitude was consistent across the track (Fig. 1b). A sound engineer (JHG) adjusted the gain of each clip in Logic Pro X (version 10.4.8, Apple Inc., Los Altos, CA, USA) to level the sound so that each segment and the full five-minute track was at the appropriate amplitude (RMS level) for each treatment (50dBA, 60dBA, or 70dBA; see spectrograms of tracks in Supplementary Fig. 1 and Supplementary Files 1, 2, 3 and 4). Noise treatments were broadcast to crickets living inside of the incubators 24 h a day (including during the daily peaks of communication by song), mimicking noise that would be experienced near a highway with high traffic fluidity. We measured the amplitude of our treatments using a PCE-430 sound level meter and class 1 microphone at 1 m away from the speaker inside of the Percival incubators. Every two weeks we confirmed the amplitude of the traffic noise tracks.

From October 2020 to August 2021, we isolated crickets just prior to their 2nd instar (~ 14 days after hatching, as early as was possible without causing mortality; following [50]) from a lab-reared population that was originally collected in Hilo, Hawaii in 2017. We randomly assigned them to one of the four traffic noise treatments (juvenile noise treatment) and housed them in groups of 15 in 1.89 L boxes inside of the incubators for the duration of their developmental exposure. Each 1.89 L box contained wet cotton for water, egg carton for shelter, and *ad libitum* Flukers Cricket Chow for food. We did this eight times over the course of eight months, yielding eight cohorts of crickets. For each cohort, we isolated 4–7 boxes of crickets per treatment, depending on the number of animals available in the lab colony, for a total of 3060 crickets assigned to treatments.

We provided clean housing and fresh food and water twice per week. To ensure that animals remained unmated prior to mating trials (see below), we isolated them individually in 0.47 L deli cups when they approached eclosion (during the final instar before adulthood) with water, shelter and food (*ad libitum* Kaytee rabbit chow). We checked the deli cups twice weekly for eclosion. At eclosion we randomly reassigned individuals to one of the four noise treatments (adult noise treatment; Fig. 1a). Throughout the experiment, we changed the stacking order of the boxes within incubators once a week (to account for the possibility that crickets would experience different loudness of noise depending on their location within the incubator) and rotated treatments among incubators every two weeks to avoid incubator effects.

### Assay overview

To measure a comprehensive set of fitness-related traits following developmental and/or adult exposure to different noise levels, we used the following general workflow: (1) at eclosion we determined development time, survival to eclosion, and measured pronotum width; (2) two weeks later we assessed mating success in controlled mating trials, allowed females to lay eggs for one week, and measured male sperm viability; (3) following the sperm viability assay or week of egg laying, we moved the crickets into the immune assays; (4) finally, when all other assays were complete, we dissected out and weighed the reproductive organs of all males and females and determined survival from eclosion to dissection (Fig. 1c).

#### Life history trait assays

When we assigned newly eclosed adults to their adult noise treatments, we measured the width of their pronotum to the nearest mm using digital calipers (assay: *pronotum*, n = 454) as a metric of adult size [56] and recorded the date on which they eclosed. To obtain a metric of development time, we subtracted the date at which they were assigned to treatments from the date of eclosion. Thus, development time here is from treatment assignment (0–14 days after hatching) to adulthood, as in [50] (assay: *development time*, n = 518). Survival to eclosion is the percent of crickets that lived to eclosion of the original 3060 crickets assigned to treatments (assay: *juvenile survival*, n = 3060), whereas the survival to dissection describes the percent of survivors from eclosion to the end of the experiment (assay: *adult survival*, n = 518).

#### Reproductive investment assays

### Mating trials

To determine whether experience with noise impacts the likelihood of individuals mating, we conducted standardized no-choice mating trials. We allowed each individual to mate only once because mating history is associated with several other fitness metrics we measured, including longevity [50], immune response [57], and reproductive organ size [58]. Whenever possible, we paired males and females from the same adult treatment for mating, but because the sexes eclosed at different rates, this was not always possible. In these cases, we paired the focal cricket with a mate pulled randomly from the large, freely breeding laboratory colony (51% of trials; whether the mate was pulled from the breeding colony or the experimental group is included as a covariate in the model). Each mating trial consisted of placing the male and female together inside of a deli cup (9 cm diameter) at ambient room temperature under dim light for up to two hours each day for up to four days (or until a single successful mating took place). We checked deli cups every 15 min for successful mating as evidenced by spermatophore transfer. After the crickets mated, or four days of unsuccessful mating trials, we again isolated the males and females in separate deli cups. Not all individuals successfully mated in the time allotted, so we were able to assess mating success (mated or not mated in four opportunities) (assay: *mated*, n = 445).

### Number of hatchlings

Next, we placed all females who mated successfully in a deli cup with food, shelter, and moist cotton and allowed them to lay eggs for one week. We then placed each female’s cotton pad in a labeled Tupperware container, providing food and shelter when hatchlings appeared. Not all females that mated had offspring hatch, allowing us to assess hatching success (zero live hatchlings or more than zero live hatchlings) (assay: *hatching success*, n = 178). Five weeks after placing the cotton pad in the Tupperware, we counted the number of offspring (assay: *hatchlings*, n = 138).

### Sperm viability

The ratio of live to dead sperm in the spermatophore is a strong predictor of male fitness in the Pacific field cricket and is highly plastic [59, 60]. Following successful mating trials, we isolated males for sperm viability analysis the following day. If males did not mate in the four days (n = 18/243, 7.4%) or if we were not able to conduct the assay the exact day after mating, we standardized time since spermatophore production by manually removing their spermatophore (externally with no injury to the cricket) 24 h before their sperm viability assay. We used a THERMOFisher LIVE/DEAD sperm viability kit to stain live and dead sperm following the protocol outlined in [60] (assay: *sperm viability*, n = 167). Briefly, we removed a spermatophore non-invasively from the male cricket, placed it on a glass slide in Beadle saline, and cut it open with dissection scissors to evacuate the sperm. We pipetted 5μL of the sperm and Beadle saline mixture to a clean part of the glass slide and gently mixed it with a sterilized pin. Then, we stained the sperm with SYBR-14 and propidium iodide (each addition followed by a 10-minute incubation period in the dark) and photographed the sperm with a Leica M165FC scope outfitted with an EC3 camera on a computer running LAS X imaging software. The GFP3 (blue) and DSR (green) fluorescent filters allowed us to take pictures of live (green) and dead (red) sperm, respectively, from the same view window on each sample.

We then crowdsourced the live and dead sperm counting using the world’s largest community science platform Zooniverse ([61]; https://www.zooniverse.org/projects/marywestwood/the-cricket-wing). We divided each sperm image into 36 smaller images and uploaded the resulting 12,304 images to our Zooniverse project. We developed a detailed protocol and training tutorial that taught volunteers to identify sperm cells and click on each cell in an image that either showed live (green) or dead (red) sperm. Volunteers were not aware of the crickets’ noise treatment, and we had 784 volunteers count sperm cells. Clicks were automatically counted and deposited in a spreadsheet accessible to the researchers. Each image was counted 6 times, a number we arrived at after visualizing the accuracy of a given image’s count after each additional replicate (after an image has been counted once, twice, thrice, etc. up to 10 replicates; see Supplementary Fig. 2). We also scanned for errors in all data caused by such issues as volunteers submitting highly inaccurate or empty counts, and removed these from the dataset. Next, we calculated the mean number of sperm in each image and identified outlier images (those below the 1st quartile or above the 3rd quartile); we did not find that certain individual volunteers regularly submitted counts that were identified as outliers. Thus, the “wisdom of the crowd” offset any one-off outlier counts. After removing erroneous counts, each image was counted an average of 5.2 +/- 0.98 times. Finally, to calculate sperm viability, we summed the counts for each male cricket across the 36 images and divided the number of live sperm by the number of dead sperm, a standard proxy for sperm quality [59].

### Reproductive organs

We dissected male and female reproductive organs after the completion of all other assays. We froze crickets at -20 °C for 15 min to euthanize them, then dissected them under a dissecting scope (Wild Heerbrugg M3Z) to extract and weigh reproductive organs. For males we removed the testes, spermatophore mold, and accessory glands (assays: *testes*, n = 158, *spermatophore mold*, n = 149, *accessory gland*, n = 157). For females we removed the ovaries (assay: *ovaries*, n = 196). We weighed each organ type separately on a VWR-64B scale immediately after dissection. Females were 33.3 +/- 9.4 days post eclosion and males were 34.8 +/- 13.8 days post eclosion at the time of dissection (age is included as a covariate in the reproductive organ models; see below).

#### Immunity assays

We assessed cricket immunity using hemocyte counts and melanization. More hemocytes indicate a stronger immune response [62]. We conducted the hemocyte counts after sperm viability (males) or one week of egg laying (females) (average age: 25 +/- 3.8 days post eclosion (females) and 23.9 +/- 5.4 days post eclosion (males)). We counted the numbers of two different types of hemocytes, plasmatocytes and granulocytes, following [63]. We placed the crickets at 5 °C for two minutes to anesthetize them, and then poked the cricket’s pronotum using a sterilized pin and pipetted 2μL of the hemolymph that emerged into 4μL of an anticoagulant buffer [64]. We then pipetted 5μL of the mixture onto a Weber Scientific Hemocytometer and placed it under a Keyence VXH Digital Microscope with a Keyence VH-Z100UR/W/T lens. We imaged the 5 × 5 grid of the hemocytometer at 400X and employed the Zooniverse platform again to count the number of each hemocyte type (on average 10 counts per image; assay: *granulocytes*, n = 213; *plasmatocytes*, n = 212).

Immediately following the hemocyte assay, we inserted a 3 mm long piece of nylon monofilament fishing line with a knot tied at the end into the previously created hole in the cricket’s pronotum. This served as a proxy for a foreign body, which induced an immune response in the crickets, allowing us to assess the level of melanization that occurred when hemocyte cells encapsulated the foreign body [65]. We left the filament in the crickets for 24 h (following [66]), then removed and imaged the filaments under a Keyence VHX Digital Microscope (Keyence Corporation, Itasca, IL USA) scope with a Keyence VH-Z20R/W/T lens at 50x magnification. We used the GNU Image Manipulation Program (v 2.10) to measure the amount of melanization on the filaments (assay: *melanization*, n = 359)—a larger proportion of melanized area indicates a stronger immune response [65].

### Statistical analyses

We performed all statistical analyses in R (v2022.12.0 + 353; [67]). First, we visualized histograms and q-q plots and determined the best distribution fit for each variable (see accompanying code and data); all variables fit the assumptions of a normal distribution. We then ran linear mixed-effect models to test whether the continuous fitness traits we measured depended on juvenile and/or adult noise treatment and generalized linear mixed-effect models for the four binomial traits (juvenile survival, mated or not, successful hatching or not, and adult survival). The basic model structure included juvenile treatment (silent, 50dBA, 60dBA, or 70dBA), adult treatment (silent, 50dBA, 60dBA, or 70dBA), and their interaction as main effects; the interaction allowed us to determine whether plasticity is stage dependent. For *pronotum, development time*, and *juvenile survival*, juvenile noise treatment (silent, 50dBA, 60dBA, or 70dBA) was the only main effect because the crickets had not experienced the adult noise treatment at the time of the assays. All models included the cohort blocking variable as a random effect. We Bonferroni corrected all *p*-values to account for the number of models run.

We included several appropriate covariates, and these differed across models. For *pronotum, development time*, and *adult survival*, we included sex as a covariate because these measures can differ between males and females [68]. The *adult survival* model also included mating success as mating status can impact longevity [50]. The binomial model addressing whether crickets *mated* or not also included sex as a covariate as well as whether the cricket they were paired with was from the experimental treatment or from the breeding colony. Both the binomial model assessing mated females’ *hatching success* and the linear model investigating the *number of hatchlings* included pronotum size and age at the end of the egg laying period as covariates. We included two covariates in the model assessing *sperm viability*, pronotum size and age at the time of the assay. The model structures for all reproductive organs included pronotum size and age at dissection as covariates, as larger crickets tend to have larger organs [54], and age can affect mass [69]. In the reproductive organ models, we also included whether the cricket successfully mated or not as a covariate, as mating may generate differences in reproductive organ mass. For the *ovary* model, we also included the number of hatchlings as a covariate. Finally, for both *hemocyte* models (number of *granulocytes*, number of *plasmatocytes*) and the *melanization* model, the covariates were pronotum size, mating success, and age at the time of the assay.

## Results

We found that juvenile traffic noise treatment impacted the percent of crickets that survived to eclosion (*juvenile survival*; *X*^*2*^ = 16.182, df = 3, *p* = 0.001)), and the interaction between a female’s traffic noise experience during juvenile and adult life stages affected her reproductive success (*number of hatchlings*; *X*^*2*^ = 319.626, df = 9, *p* < 0.001). However, the consistent traffic noise treatments (50dBA, 60dBA, 70dB) did not impact the other 13 fitness-related traits we investigated, irrespective of noise level and whether traffic noise was experienced by crickets during development only, at adulthood only, or during both life stages (Tables 1 and 2).

**Fig. 2:**
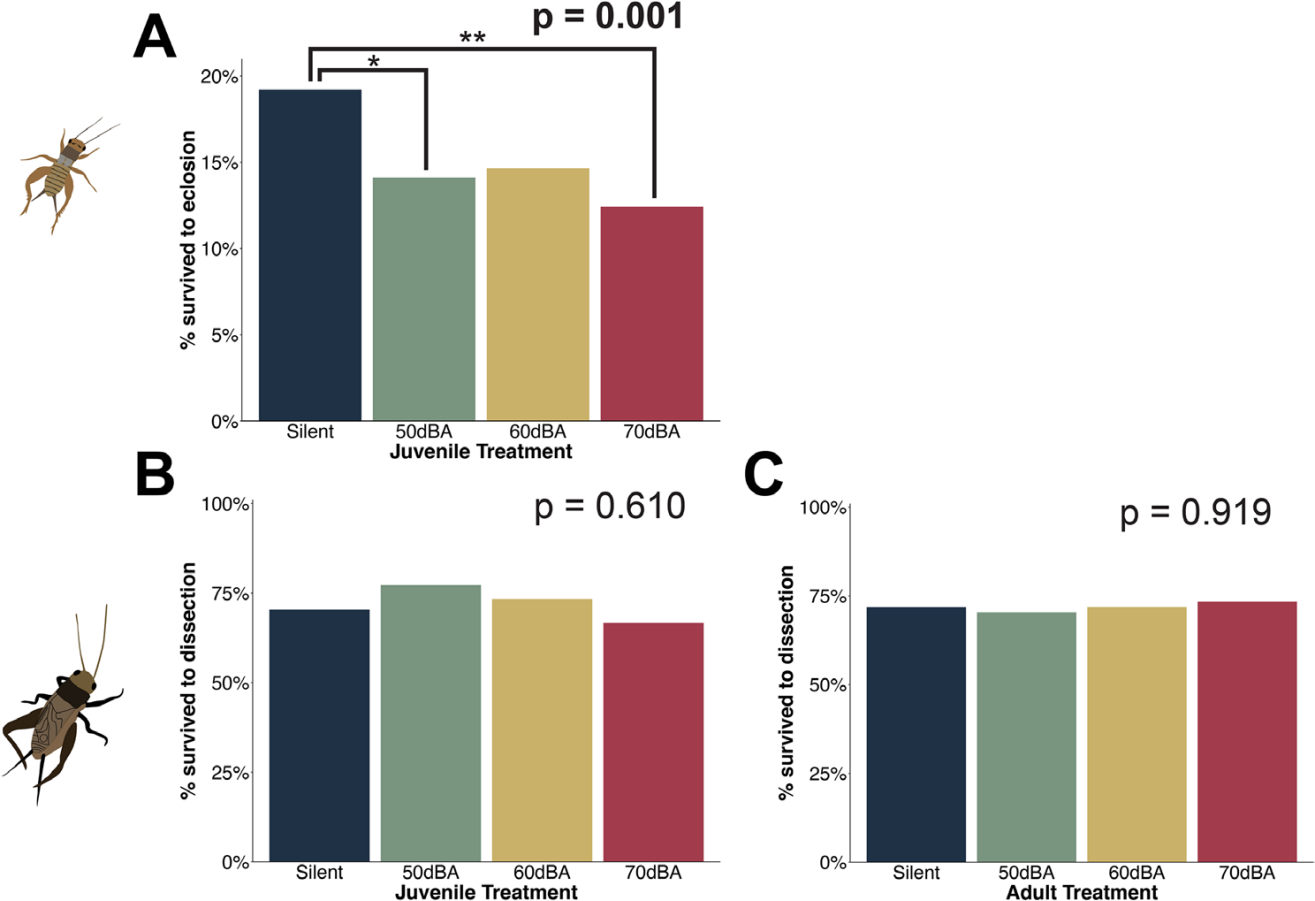
Impacts of juvenile and adult noise exposure on survival. Juvenile noise treatment impacted the percent of crickets that survived to eclosion (*juvenile survival*, Panel **A**), but not the percent of adult crickets that made it to the final assay, dissection (*adult survival*, Panel **B**). Adult noise treatment also did not affect *adult survival* (Panel **C**)

**Fig. 3:**
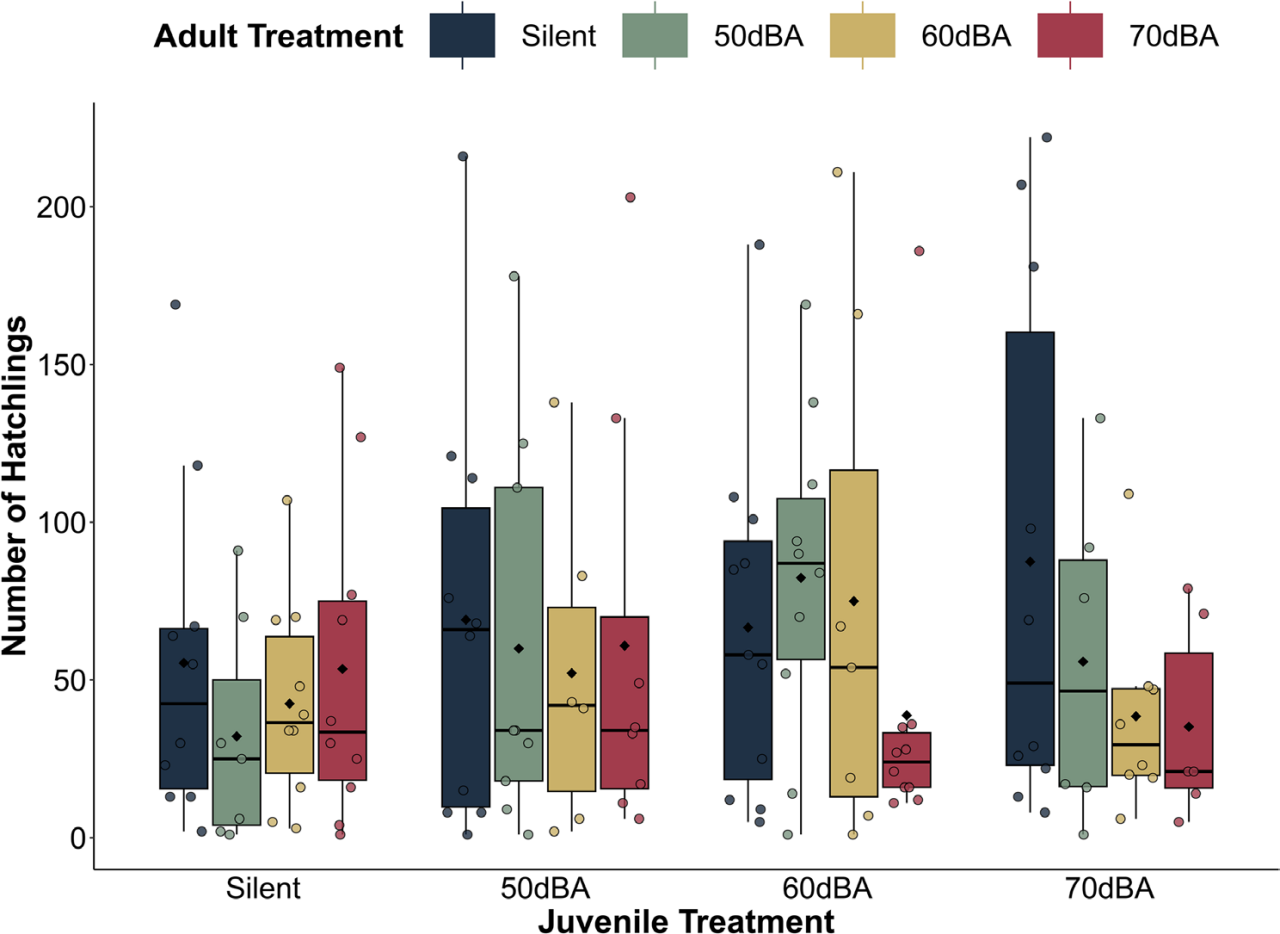
Interactive impacts of juvenile and adult noise on number of hatchlings. The number of live hatchlings five weeks after egg laying ended depended on the interaction between a female’s juvenile (x axis) and adult (boxplot colors) noise treatment (for females that had at least one hatchling). The box displays the first quartile (bottom), median (horizontal line), and third quartile (top) of the data. The lower and upper whiskers denote the minimum and maximum of the data, respectively

The main effect of noise experienced during development was such that juvenile crickets reared in silence had higher survival to eclosion (19.1%) than crickets reared in 50dBA (14.1%; *z* = -2.782, *p* = 0.028) and 70dBA (12.4%; *z* = -3.77, *p* = 0.001), but not significantly higher than those reared in 60dBA (though the trend was in that direction; 14.6%, *z* = -2.485, *p* = 0.062) (Fig. 2). The interactive effect of juvenile and adult noise treatments on number of hatchlings was rather complex (Fig. 3) and supports the hypothesis that there are stage-dependent effects of noise that differ depending on the specific characteristics of the noise (in this case amplitude) and that the impacts of experience at one life-stage may depend on experience at other life-stages. A detailed account of the many contrasts contributing to this effect can be found in Supplementary Tables 1 and Supplementary Fig. 3. After Bonferroni correction, other life-history characteristics like *development time*, *pronotum* (size at adulthood), and *adult survival* did not depend on noise experience or levels, nor did reproductive traits like the likelihood of *mating*, *hatching success*, the mass of *reproductive organs* (ovary, spermatophore mold, testes, and accessory gland), or *sperm viability*. Likewise, the immunity traits we measured (*melanization, plasmatocyte* counts, and *granulocyte* counts) did not depend on experience with noise (after Bonferroni correction). We provide plots showing main effects that were significant before, but not after, the Bonferroni correction (before: α = 0.05, after: α = 0.003) in the supplementary materials (Supplementary Fig. 4). Moreover, the random effect in our models, cohort, only contributed substantial variation to our model investigating *development time* (Supplementary Table 2). We discuss why there may be differences in development time across cohorts in Supplementary Figs. 5–6 and Supplementary Table 3.

While our noise treatments did not impact the majority of measured fitness traits, we did find some additional relationships between covariates (i.e., sex, size, and age) and measured fitness traits that remained significant after Bonferroni correction. For instance, we found that *development time* differed between the sexes; on average females developed 7.3 days faster than males (females: 94.3 days, males: 101.6 days; *X*^*2*^ = 74.403, df = 1, *p* < 0.001; Supplementary Fig. 7). It is perhaps unsurprising, then, that we also found that females are 5.4% smaller than males, given that insect size and development time are generally related [70] (females: 5.3 mm, males: 5.6 mm; *X*^*2*^ = 104.628, df = 1, *p* < 0.001; Supplementary Fig. 8). As for reproductive investment, females that mated successfully were more likely to have at least one hatchling if they were larger (*X*^*2*^ = 9.751, df = 1, *p* = 0.002) and, of females that had at least one offspring, those that were older at the time of egg laying had more hatchlings (*X*^*2*^ = 34.116, df = 1, *p* < 0.001; Supplementary Fig. 9). As for males, larger crickets had smaller testes masses (*X*^*2*^ = 12.396, df = 1, *p* < 0.001; Supplementary Fig. 10). And, age at the time of dissection impacted both testes and ovary masses: older females had larger ovaries (*X*^*2*^ = 13.940, df = 1, *p* < 0.001), whereas younger males had larger testes (*X*^*2*^ = 80.589, df = 1, *p* < 0.001; Supplementary Figs. 11–12).

**Table 1:** Results from Generalized Linear Mixed Models

	Model Name & Type	Parameter	(LR) Chisq	Df	Pr(> Chisq)
**A. Life History**					
	**Development time**				
		juvenile treatment	5.998	3	0.112
		**sex**	**74.403**	**1**	**< 0.001**
	**Pronotum (mm)**				
		juvenile treatment	5.708	3	0.127
		**sex**	**104.628**	**1**	**< 0.001**
	**Juvenile Survival**				
		**juvenile treatment**	**16.182**	**3**	**0.001**
	**Adult Survival**				
		juvenile treatment	1.821	3	0.61
		adult treatment	0.5	3	0.919
		sex	4.67	1	0.031
		mated	8.266	1	0.004
**B. Reproductive Investment**					
	**Mated**				
		juvenile treatment	6.08	3	0.108
		adult treatment	3.115	3	0.108
		juvenile*adult treatment	7.649	9	0.57
		sex	7.568	1	0.006
		pair status	0.246	1	0.62
	**Hatching Success**				
		juvenile treatment	1.292	3	0.731
		adult treatment	2.426	3	0.33
		juvenile*adult treatment	6.972	9	0.64
		pronotum	9.751	1	0.002
		age	0.012	1	0.914
	**Number of Hatchlings**				
		**juvenile treatment**	**39.496**	**3**	**< 0.001**
		**adult treatment**	**40.83**	**3**	**< 0.001**
		juvenile*adult treatment	**319.627**	**9**	**< 0.001**
		pronotum	5.66	1	0.017
		**age**	**34.118**	**1**	**< 0.001**
	**Sperm viability**				
		juvenile treatment	0.268	3	0.966
		adult treatment	0.194	3	0.979
		juvenile*adult treatment	2.705	9	0.975
		pronotum	0.017	1	0.895
		age	0.032	1	0.858
	**Ovary mass (g)**				
		juvenile treatment	1.107	3	0.775
		adult treatment	0.109	3	0.991
		juvenile*adult treatment	7.427	9	0.593
		mated	-	0	-
		pronotum	5.892	1	0.015
		number of hatchlings	0.126	1	0.722
		**age**	**1394**	**1**	**< 0.001**
	**Testes mass (g)**				
		juvenile treatment	0.536	3	0.911
		adult treatment	0.622	3	0.891
		juvenile*adult treatment	6.311	9	0.708
		mated	0	1	0.999
		**pronotum**	**12.396**	**1**	**< 0.001**
		**age**	**80.589**	**1**	**< 0.001**
	**Accessory gland mass (g)**				
		juvenile treatment	1.492	3	0.684
		adult treatment	1.539	3	0.673
		juvenile*adult treatment	3.531	9	0.939
		mated	0.33	1	0.566
		pronotum	2.247	1	0.134
		age	7.297	1	0.007
	**Spermatophore mold mass (g)**				
		juvenile treatment	1.526	3	0.676
		adult treatment	1.383	3	0.71
		juvenile*adult treatment	4.591	9	0.868
		mated	1.595	1	0.207
		pronotum	0.753	1	0.386
		age	0.407	1	0.524
**C. Immunity**					
	**Granulocytes**				
		juvenile treatment	6.242	3	0.1
		adult treatment	2.598	3	0.458
		juvenile*adult treatment	10.296	9	0.327
		mated	2.203	1	0.138
		pronotum	0.289	1	0.591
		age	5.061	1	0.024
		sex	0.002	1	0.962
	**Plasmatocytes**				
		juvenile treatment	8.946	3	0.03
		adult treatment	5.378	3	0.146
		juvenile*adult treatment	15.353	9	0.082
		mated	0.825	1	0.364
		pronotum	0.065	1	0.799
		age	1.73	1	0.188
		sex	2.833	1	0.092
	**Melanization**				
		juvenile treatment	3.917	3	0.271
		adult treatment	2.477	3	0.48
		juvenile*adult treatment	6.745	9	0.664
		mated	0.414	1	0.52
		pronotum	6.145	1	0.013
		age	0.001	1	0.971
		sex	5.922	1	0.015

Results from (Generalized) Linear Mixed Models (cohort = random effect) assessing the effects of different levels of anthropogenic noise on fitness-related life-history (A), reproduction (B), and immunity (C) traits. Bold indicates significant results after Bonferroni adjustment, α = 0.0033. The *ovary* model dropped the mated coefficient because the fixed-effect model matrix was rank deficient

## Discussion

Plastic responses may allow organisms to survive and reproduce in rapidly changing urban environments. Within-generation plasticity, whether triggered during development or at adulthood, sometimes allows populations to persist long enough in novel environments for adaptation to occur [14]. In the Pacific field cricket, previous studies found that exposure to very loud traffic noise containing frequent changes in amplitude during development and/or chronically throughout life induced plastic changes in some behavioral and life-history traits. Table 2 summarizes this work and compares previous findings to findings in this manuscript [46, 50, 58]. We expanded upon these previous studies to investigate the impacts of relatively consistent noise broadcast at amplitudes commonly experienced by animals living in suburban and urban environments during development or adulthood on a broader suite of traits. We found that two of the fifteen fitness-related traits we investigated were significantly impacted by noise exposure, suggesting that noise was a stressor, particularly during the juvenile stage. A greater percent of juvenile crickets survived to eclosion in the silent treatment than the noise treatments. The number of live offspring hatched also depended on the interaction between a female’s juvenile and adult exposure to traffic noise, though the result here was rather complex. Given previous findings in this species, we were initially surprised to find little evidence of within-generation changes in fitness-related traits in response to a noise stressor, even at the highest amplitude tested (70dBA; Table 2). However, upon careful consideration of differences between this study and previous work, we speculate that this could be due to habituation to the noise, and/or specific characteristics of the noise that differ from those previously tested. For instance, plasticity may be better able to buffer potential negative impacts of noise when the noise is relatively consistent, rather than highly variable. We expand on these ideas below and consider avenues for further exploration of the fitness impacts of noise.

**Table 2:** Effects of different traffic noise characteristics on *T. oceanicus*

	Trait	Consistent Noise (this paper)	Loud, Variable Noise (past research)	Citation
**A. Life History**				
	**Development time**	sex	noise	Gurule-Small and Tinghitella 2019
	**Adult lifespan**	-	noise, sex, pronotum	Gurule-Small and Tinghitella 2019
	**Adult size**	sex	sex	Gurule-Small and Tinghitella 2019
	**Juvenile survival**	noise	n.s.	Gurule-Small and Tinghitella 2019
	Adult survival*	n.s.	noise, sex, mated	Gurule-Small and Tinghitella 2019
**B. Reproductive Investment**				
	**Number of eggs**	-	n.s.	Gurule-Small and Tinghitella 2019
	**Proportion of surviving eggs**	-	n.s.	Gurule-Small and Tinghitella 2019
	Hatching success**	pronotum	-	
	Number of hatchlings**	noise, sex, pronotum	n.s.	Gurule-Small and Tinghitella 2019
	**Ovary mass**	age	-	
	**Testes mass**	age, pronotum	n.s.	Bowen et al. 2020
	**Accessory gland mass**	n.s.	-	
	**Spermatophore mold mass**	n.s.	noise	Bowen et al. 2020
	**Body mass**	-	n.s.	Bowen et al. 2020
**C. Behavior**				
	Mated***	sex	n.s.	Gurule-Small and Tinghitella 2019
	**Time to first movement**	-	noise	Gurule-Small and Tinghitella 2018
	**Time to contact speaker**	-	noise	Gurule-Small and Tinghitella 2018
	**Search time**	-	-	Gurule-Small and Tinghitella 2018
	**Number of grids crossed**	-	pronotum	Gurule-Small and Tinghitella 2018

Summary of findings in this study compared to those from previous studies in the Pacific field cricket in which variable, but loud traffic noise was broadcast. There are other additional differences in experimental design that may also contribute to the differences we see here. For instance, all previous investigations were conducted with crickets from Mo’orea, French Polynesia, whereas here we study animals from Hilo, Hawaii. Gurule-Small and Tinghitella (2018) used a similar design to this experiment, exposing animals to noise during development, adulthood, both, or neither, but Gurule-Small and Tinghitella (2019) exposed the crickets to noise from the penultimate instar through adulthood, and Bowen et al. (2020) exposed the crickets to noise beginning at the 2nd instar and throughout adulthood

* Note that *adult survival* was measured slightly differently in the two studies. Our design required dissecting individuals within a standard age range, so we calculated adult survival as the proportion of adults that survived to dissection, whereas Gurule-Small and Tinghitella (2019) measured the number of days adults lived until natural death

** We counted the *hatching success* (whether a female had zero live hatchlings or more than zero live hatchlings) and *number of hatchlings* 5 weeks after the female’s week of egg laying ended, whereas Gurule-Small and Tinghitella (2019) counted the total number of eggs laid and the number of eggs that hatched into the first instar. In addition, Gurule-Small and Tinghitella (2019) recorded the number of eggs over the course of a female’s lifetime, whereas we collected eggs for only 1 week, therefore the absolute number of hatchlings are not directly comparable between experiments, though patterns of survival are

***We conducted the *mated* assay with both sexes, while Gurule-Small and Tinghitella (2019) only conducted it in females

We found a significant impact of juvenile treatment on the percent of crickets that survived to eclosion. A greater proportion of crickets reared in silence survived to eclosion than other noise treatments (19% survival in silent treatment versus ~ 12 to 15% survival in noise treatments; Fig. 2), though this relationship was not strictly statistically significant when compared to the 60dBA treatment (*p* = 0.062). Adult noise exposure did not impact survival during that stage of life (Fig. 2). Taken together, these results support our hypothesis that the effects of noise on fitness are stage-dependent. Limited research has been conducted on noise impacts during development, but existing studies have shown that noise disrupts scallop larval development [71], and some of the mortality induced by noise in juvenile stages of tree swallows is attributed to elevated stress hormones and stress responses [31]. Our current result is especially interesting given that insect auditory organs do not develop until their penultimate instar [72], so the effect of noise on survival is likely realized during the relatively short period during which the crickets could hear the airborne traffic noise (the last ~ 2 weeks of juvenile development). This is consistent with previous work in which we found effects of noise experienced between the penultimate molt and eclosion to adulthood on mating-related behaviors [46]. It is also possible that the experimental crickets can detect and respond to substrate-borne vibration stemming from the traffic noise playback, and that substrate-borne vibration was detectable throughout their development. We placed a speaker on an upper-level wire rack within each incubator to generate each of our treatments, and presumably the three noise treatments produced vibrations. Crickets develop a functional cercal system in their first instar [72] so vibrations detected through the substrate could impact them much earlier in their development than airborne noise. Further, substrate-borne vibration is a described communication modality in this system [73, 74]. The reduction in juvenile survival under noisy conditions uncovered here could have negative consequences for population demographics and evolutionary change (smaller populations with lower genetic diversity may be less able to respond evolutionarily to the environment); these impacts are especially critical to understand for threatened or endangered insects.

The second effect of noise on fitness we found was an interaction between juvenile noise treatment and adult noise treatment on the number of hatchlings. This finding supports our hypothesis that effects of noise are stage-dependent, may vary with the specific characteristics of noise (here, amplitude), and that experiences at different life stages are not simply cumulative. However, the interaction between juvenile and adult noise exposure was rather complex (Fig. 3). We provide a detailed account of the contrasts contributing to this statistically strong effect in the supplementary material, but hesitate to overinterpret the contrasts because the overall sample is limited to only females who had at least one live hatchling, and after parsing across the 16 treatment groups, the sample size is relatively small.

The lack of an effect of noise treatments on most of the fitness-related traits we investigated could indicate that (1) noise truly does not impact the measured traits or (2) plasticity effectively buffered the effects of noise. Alternatively, (3) perhaps the crickets became habituated to the noise or (4) the noise treatment was too consistently stressful to yield plastic responses without incurring very high costs. An important qualification to the first explanation that noise does not impact fitness traits is that the nature of the traffic sound characteristics may be particularly relevant. Continuous stressors often have different impacts on organismal stress responses than variable or unpredictable stressors. For instance, studies have shown that intermittent noise impacts some traits that continuous noise does not; these traits include acute stress responses in giant kelpfish [75], lek abundance in sage grouse [76], and behavioral recovery times in European seabass [77]. Additionally, a meta-analysis revealed that noises with irregular frequencies and/or amplitudes caused the most stress and negative reproductive outcomes in fish [78]. To test whether the amplitude of noise experienced during development or at adulthood impacted plastic responses of crickets, we first had to level the tracks such that there was increased consistency in the sounds broadcast (Fig. 1b). Otherwise, the amplitude would have varied substantially within noise treatments (as in the original track used in [46, 50]; see Fig. 1b). As a consequence, this leveling led to less variable, and perhaps more predictable, playbacks than were used in previous studies in the same species and may explain why some fitness traits, development time and spermatophore mold mass, were impacted by noise in a previous study but not in ours (Table 2; [46]). Under the second potential explanation, the louder noise levels may have initially negatively impacted the crickets’ fitness-related traits, but through plasticity, they may have been able to compensate leading to no observable differences among the treatments.

Alternatively, the regularity and chronic nature of this study’s traffic noise playbacks could have allowed the animals to habituate more easily to the sound and alleviated some of the negative fitness impacts found in previous studies. Habituation is the decrease in a response as a result of repeated stimulus exposure [79] and is thought to help animals to minimize predation risk and avoid energetic responses to harmless stimuli [80, 81]. Insects are well-known to habituate to many stimuli [82]. In this scenario, our results may indicate that animals are able to “tune out” constant, relatively uniform stimuli. Such a process might facilitate survival in some urban environments. Finally, the literature suggests that there is a point at which plastic responses to stressors become too costly [83, 84]. For example, the predator risk allocation hypothesis suggests that in environments where risks are consistently high, animals should devote fewer resources towards avoiding those risks than they should at intermediate risk levels [85]. A similar phenomenon could be occurring here. The chronic (played constantly throughout development, adulthood, or both) noise exposures we used may have been too consistently stressful to yield plastic responses without incurring very high costs, even at our lower amplitudes.

In addition to the previously mentioned possible explanations for differences between our work and previous work on noise in crickets (no effect of noise on measured traits, effective plasticity, habituation, or insurmountable fitness costs), previous studies investigated behavioral responses to traffic noise, while we focused on life history, reproduction, and immunity-related traits. For example, work in other cricket species revealed that anthropogenic noise limited mate searching behaviors [51]. Likewise, in *T. oceanicus*, most of the significant noise treatment impacts were found in mate searching behaviors; mate location was hindered by development in loud traffic noise (70dBA; [45]) and in previous work, researchers found that the crickets had particularly low mating rates under 70dBA traffic noise (Table 2; [50]). Taken together, this indicates that noise exposure during development or adulthood may be particularly consequential for behavioral traits relevant to mating, though further investigation is necessary to parse out exactly which behavioral aspects. Here, we did not find that noise experience impacted whether or not crickets mated (but in this study, as is standard for work in this species because of the low mating rates under noise described above and in [50], mating assays took place in ambient noise not under noise treatments). In another cricket system, courtship behavior was largely unaffected by traffic noise played during mating trials [52]. As such, these results suggest that in nature, noise experienced could have a large impact on these crickets’ abilities to find each other and may impact their mating success. It is also worth noting that previous work on the effects of traffic noise on fitness in *T. oceanicus* was conducted using a population from Mo’orea, French Polynesia [46, 50], whereas the current study was conducted with a population from Hilo, Hawaii. It is possible that population-level differences contribute to the differences in effects of noise that we find across studies.

Finally, it is important to acknowledge that we did not directly measure stress in this study. In vertebrates, anthropogenic noise can significantly increase levels of so-called stress hormones like glucocorticoids [31, 32, 86, 87]. However, insect stress responses are not as well-studied. While the negative impacts we uncovered on survival suggest our treatments were perceived as stressors, directly measuring insect stress responses would be interesting to do in future work. Additionally, related to the habituation discussion above, our noise exposures may have initially induced stress responses that subsided over time. This area of research deserves additional attention, particularly when coupled with less predictable, more variable noise treatments.

## Conclusion

Overall, after a robust evaluation of the effects of anthropogenic noise on numerous fitness traits, we found that the majority of measured traits (13 out of 15) were not affected by traffic noise with a consistent amplitude, regardless of when that noise was experienced or how loud it was. This may indicate that plasticity is sufficient to buffer potentially negative consequences, that animals may habituate to consistent anthropogenic noise conditions, or indeed that anthropogenic noise does not have an effect at all on some of these traits (but see Table 2). Future studies should carefully consider the characteristics of acoustic stressors (e.g. consistency, ecologically relevant anthropogenic noise), as these may impact the effects uncovered, and thus our understanding of how commensal animals survive and reproduce in human-adjacent communities. Further, testing unmeasured effects, such as stress responses, and performing such studies in the field would significantly add to our understanding of the realized fitness consequences of anthropogenic noise exposure. Our result that juvenile survival under noisy conditions is reduced is especially relevant for conservation and management decisions, and future work should clarify consequences in natural settings with varying noise characteristics and the generalizability across insects.

### Electronic supplementary material

Below is the link to the electronic supplementary material.

**Supplementary Material 1:** **Silent_5min_in_incubator.** 5-minute recording of the entire traffic track played in the silent treatment incubator. The recording was taken from 1m away from the speaker inside of the closed incubator

**Supplementary Material 2:** **50dBA_5min_in_incubator.** 5-minute recording of the entire traffic track played in the 50dBA treatment incubator. The recording was taken from 1m away from the speaker inside of the closed incubator

**Supplementary Material 3:** **60dBA_5min_in_incubator.** 5-minute recording of the entire traffic track played in the 60dBA treatment incubator. The recording was taken from 1m away from the speaker inside of the closed incubator

**Supplementary Material 4:** **70dBA_5min_in_incubator.** 5-minute recording of the entire traffic track played in the 70dBA treatment incubator. The recording was taken from 1m away from the speaker inside of the closed incubator. See attached “.wav” file

**Supplementary Material 5:** **Figure S1.** Spectrograms for the four different noise treatments. **Figure S2.** Process for visualizing the accuracy of a given sperm viability’s count. **Table S1.** Full output of pairwise comparisons comprising the interactive effect of juvenile and adult noise treatment on the number of hatchlings. **Figure S3.** Comparison arrow plots for the pairwise results of number of hatchlings. **Figure S4.** Impact of juvenile treatment on the number of plasmatocytes. **Table S2.** The amount of variance for each model that is attributed to among cohort differences. **Figure S5**. Plot of variation in development time among cohorts. **Table S3.** Full output of pairwise comparisons for the effect of cohort on development time. **Figure S6.** Comparison arrow plots for the pairwise results of the effect of cohort on development time. **Figures S7-12.** Significant relationships between covariates and measured fitness traits

## Data Availability

Data and R code files are available on Dryad (10.5061/dryad.9w0vt4bnx). No permissions are required to access the data.
