# Supplementary material for: Consistent traffic noise impacts few fitness-related traits in a field cricket
Source: BMC Ecol Evol. 2023 Dec 20;23:78. doi: 10.1186/s12862-023-02190-2 (PMC10731782; doi:10.1186/s12862-023-02190-2)
Supplement: Supplementary file 5 — Supplementary Material 5: Figure S1. Spectrograms for the four different noise treatments. Figure S2. Process for visualizing the accuracy of a given sperm viability’s count. Table S1. Full output of pairwise comparisons comprising the interactive effect of juvenile and adult noise treatment on the number of hatchlings. Figure S3. Comparison arrow plots for the pairwise results of number of hatchlings. Figure S4. Impact of juvenile treatment on the number of plasmatocytes. Table S2. The amount of variance for each model that is attributed to among cohort differences. Figure S5. Plot of variation in development time among cohorts. Table S3. Full output of pairwise comparisons for the effect of cohort on development time. Figure S6. Comparison arrow plots for the pairwise results of the effect of cohort on development time. Figures S7-12. Significant relationships between covariates and measured fitness traits [file 12862_2023_2190_MOESM5_ESM.docx]

Supplementary Information for Welsh, Anner et al. (2024) “Consistent traffic noise impacts few fitness-related traits in a field cricket” in *BMC Ecology & Evolution*


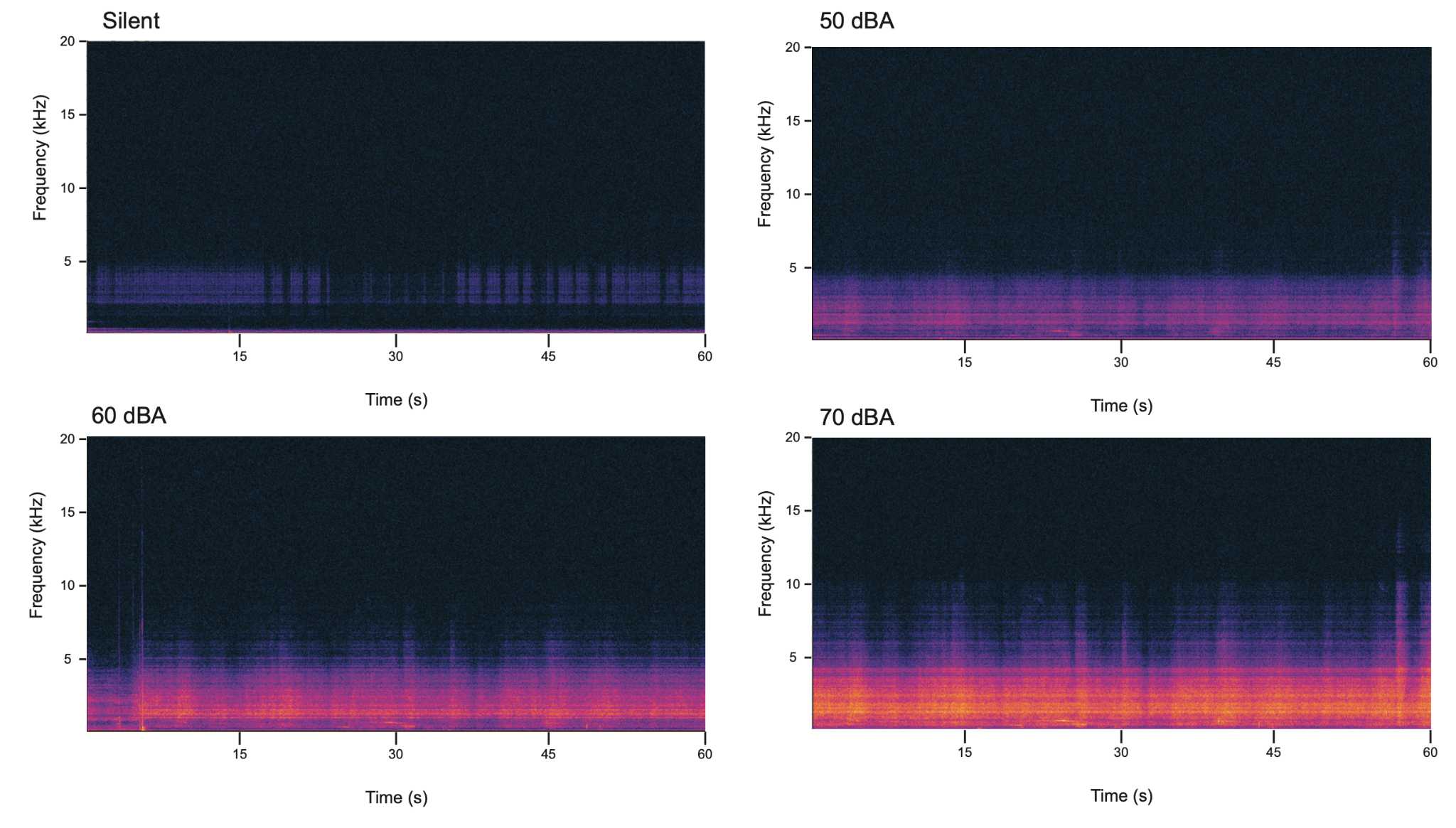


**Supplementary Figure 1.** Spectrograms for the four different noise treatments. Each shows the first minute of the 5 minute traffic track played at the associated amplitudes (silent, 50dBA, 60dBA, 70dBA).

**Supplementary_File_1_Silent_5min_in_incubator.** 5-minute recording of the entire traffic track played in the silent treatment incubator. The recording was taken from 1m away from the speaker inside of the closed incubator. See attached “.wav” file.

**Supplementary_File_2_50dBA_5min_in_incubator.** 5-minute recording of the entire traffic track played in the 50dBA treatment incubator. The recording was taken from 1m away from the speaker inside of the closed incubator. See attached “.wav” file.

**Supplementary_File_3_60dBA_5min_in_incubator.** 5-minute recording of the entire traffic track played in the 60dBA treatment incubator. The recording was taken from 1m away from the speaker inside of the closed incubator. See attached “.wav” file.

**Supplementary_File_4_70dBA_5min_in_incubator.** 5-minute recording of the entire traffic track played in the 70dBA treatment incubator. The recording was taken from 1m away from the speaker inside of the closed incubator. See attached “.wav” file.


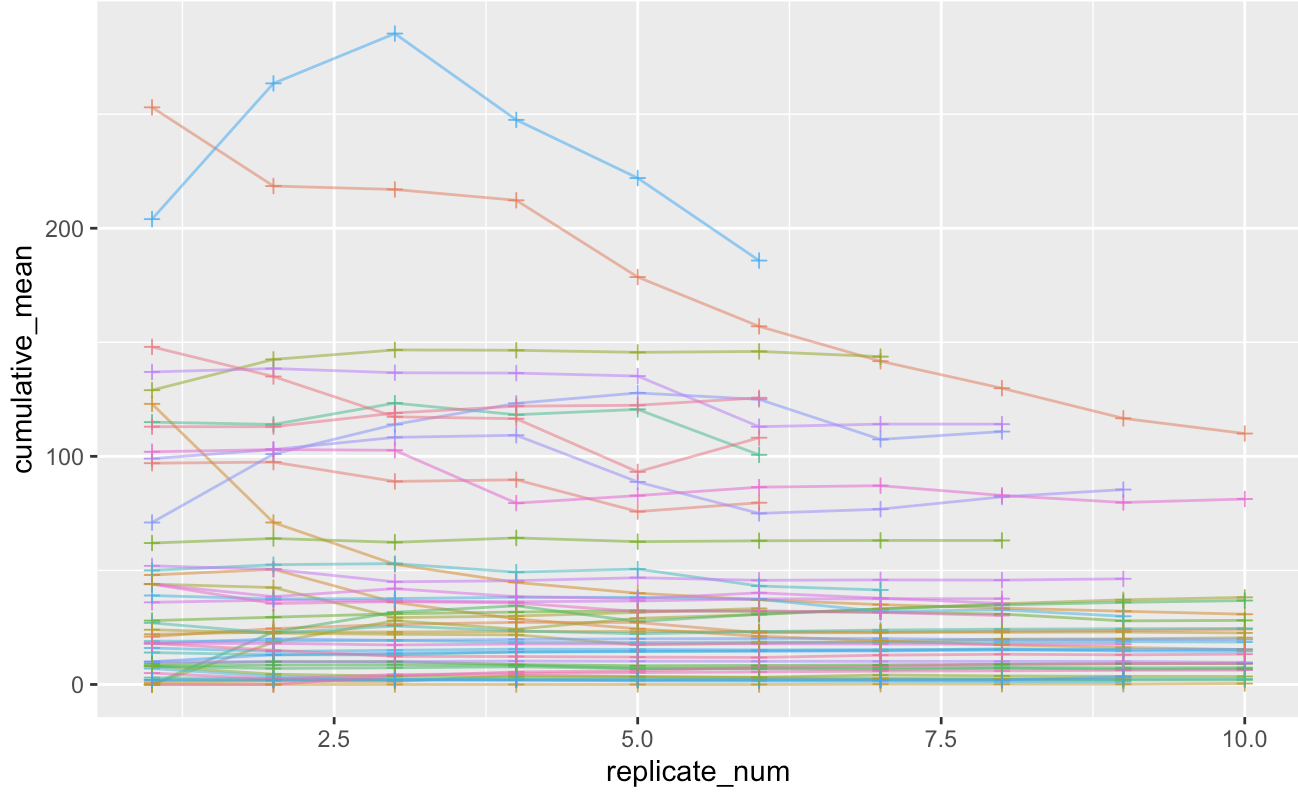


**Supplementary Figure 2**. Process for visualizing the accuracy of a given sperm viability image’s count after each additional replicate. We selected 50 random sperm counts for both live and dead sperm and visualized cumulative mean after each successive count and determined that 6 replicates was sufficient for the majority of counts.

**Supplementary Table 1.** Full output of pairwise comparisons comprising the interactive effect of juvenile and adult noise treatment on the number of hatchlings. The contrast column lists a comparison of two of the 16 treatment types. Each treatment type is written as juvenile treatment:adult treatment.

| *Simple contrasts for adult treatment* | | | | | |
| --- | --- | --- | --- | --- | --- |
| **juvenile treatment = silent** | | | | | |
| contrast | estimate | SE | df | z.ratio | p.value |
| silent:silent - silent:50 | -0.168369 | 0.0826 | Inf | -2.038 | 0.1742 |
| silent:silent - silent:60 | -0.389939 | 0.0687 | Inf | -5.68 | <.0001 |
| silent:silent - silent:70 | -0.000747 | 0.0611 | Inf | -0.012 | 1 |
| silent:50 - silent:60 | -0.221571 | 0.0845 | Inf | -2.621 | 0.0435 |
| silent:50 - silent:70 | 0.167622 | 0.0824 | Inf | 2.033 | 0.1758 |
| silent:60 - silent:70 | 0.389193 | 0.0681 | Inf | 5.713 | <.0001 |
| **juvenile treatment = 50** | | | | | |
| contrast | estimate | SE | df | z.ratio | p.value |
| 50:silent - 50:50 | 0.362714 | 0.0638 | Inf | 5.681 | <.0001 |
| 50:silent - 50:60 | -0.119969 | 0.0725 | Inf | -1.655 | 0.3479 |
| 50:silent - 50:70 | 0.032754 | 0.0629 | Inf | 0.521 | 0.9541 |
| 50:50 - 50:60 | -0.482682 | 0.0763 | Inf | -6.323 | <.0001 |
| 50:50 - 50:70 | -0.329959 | 0.0664 | Inf | -4.966 | <.0001 |
| 50:60 - 50:70 | 0.152723 | 0.0763 | Inf | 2.003 | 0.1869 |
| **juvenile treatment = 60** | | | | | |
| contrast | estimate | SE | df | z.ratio | p.value |
| 60:silent - 60:50 | -0.300032 | 0.0515 | Inf | -5.822 | <.0001 |
| 60:silent - 60:60 | -0.031153 | 0.0596 | Inf | -0.523 | 0.9536 |
| 60:silent - 60:70 | 0.708057 | 0.0672 | Inf | 10.543 | <.0001 |
| 60:50 - 60:60 | 0.268879 | 0.0576 | Inf | 4.668 | <.0001 |
| 60:50 - 60:70 | 1.008089 | 0.0662 | Inf | 15.225 | <.0001 |
| 60:60 - 60:70 | 0.73921 | 0.0716 | Inf | 10.328 | <.0001 |
| **juvenile treatment = 70** | | | | | |
| contrast | estimate | SE | df | z.ratio | p.value |
| 70:silent - 70:50 | 0.002415 | 0.0726 | Inf | 0.033 | 1 |
| 70:silent - 70:60 | 0.397294 | 0.0699 | Inf | 5.681 | <.0001 |
| 70:silent - 70:70 | 0.856265 | 0.0779 | Inf | 10.995 | <.0001 |
| 70:50 - 70:60 | 0.394879 | 0.0852 | Inf | 4.633 | <.0001 |
| 70:50 - 70:70 | 0.85385 | 0.0947 | Inf | 9.016 | <.0001 |
| 70:60 - 70:70 | 0.458972 | 0.0929 | Inf | 4.938 | <.0001 |
|  | | | | | |
| *Simple contrasts for juvenile treatment* | | | | | |
| **adult treatment = silent** | | | | | |
| contrast | estimate | SE | df | z.ratio | p.value |
| silent:silent - 50:silent | -0.31922 | 0.0616 | Inf | -5.179 | <.0001 |
| silent:silent - 60:silent | -0.32885 | 0.0593 | Inf | -5.546 | <.0001 |
| silent:silent - 70:silent | -0.14583 | 0.0571 | Inf | -2.555 | 0.0519 |
| 50:silent - 60:silent | -0.00963 | 0.0581 | Inf | -0.166 | 0.9984 |
| 50:silent - 70:silent | 0.17339 | 0.0589 | Inf | 2.945 | 0.017 |
| 60:silent - 70:silent | 0.18302 | 0.0557 | Inf | 3.283 | 0.0057 |
| **adult treatment = 50** | | | | | |
| contrast | estimate | SE | df | z.ratio | p.value |
| silent:50 - 50:50 | 0.21186 | 0.0844 | Inf | 2.511 | 0.0582 |
| silent:50 - 60:50 | -0.46052 | 0.0785 | Inf | -5.866 | <.0001 |
| silent:50 - 70:50 | 0.02495 | 0.094 | Inf | 0.266 | 0.9935 |
| 50:50 - 60:50 | -0.67237 | 0.06 | Inf | -11.2 | <.0001 |
| 50:50 - 70:50 | -0.18691 | 0.0767 | Inf | -2.438 | 0.0702 |
| 60:50 - 70:50 | 0.48546 | 0.0737 | Inf | 6.584 | <.0001 |
| **adult treatment = 60** | | | | | |
| contrast | estimate | SE | df | z.ratio | p.value |
| silent:60 - 50:60 | -0.04925 | 0.076 | Inf | -0.648 | 0.9163 |
| silent:60 - 60:60 | 0.02993 | 0.0684 | Inf | 0.438 | 0.972 |
| silent:60 - 70:60 | 0.6414 | 0.0776 | Inf | 8.269 | <.0001 |
| 50:60 - 60:60 | 0.07919 | 0.0752 | Inf | 1.052 | 0.7184 |
| 50:60 - 70:60 | 0.69065 | 0.0839 | Inf | 8.23 | <.0001 |
| 60:60 - 70:60 | 0.61146 | 0.0724 | Inf | 8.444 | <.0001 |
| **adult treatment = 70** | | | | | |
| contrast | estimate | SE | df | z.ratio | p.value |
| silent:60 - 50:60 | -0.28572 | 0.0649 | Inf | -4.401 | 0.0001 |
| silent:60 - 60:60 | 0.37995 | 0.0708 | Inf | 5.365 | <.0001 |
| silent:60 - 70:60 | 0.71118 | 0.0837 | Inf | 8.497 | <.0001 |
| 50:60 - 60:60 | 0.66567 | 0.0728 | Inf | 9.141 | <.0001 |
| 50:60 - 70:60 | 0.9969 | 0.0889 | Inf | 11.217 | <.0001 |
| 60:60 - 70:60 | 0.33123 | 0.0911 | Inf | 3.637 | 0.0016 |


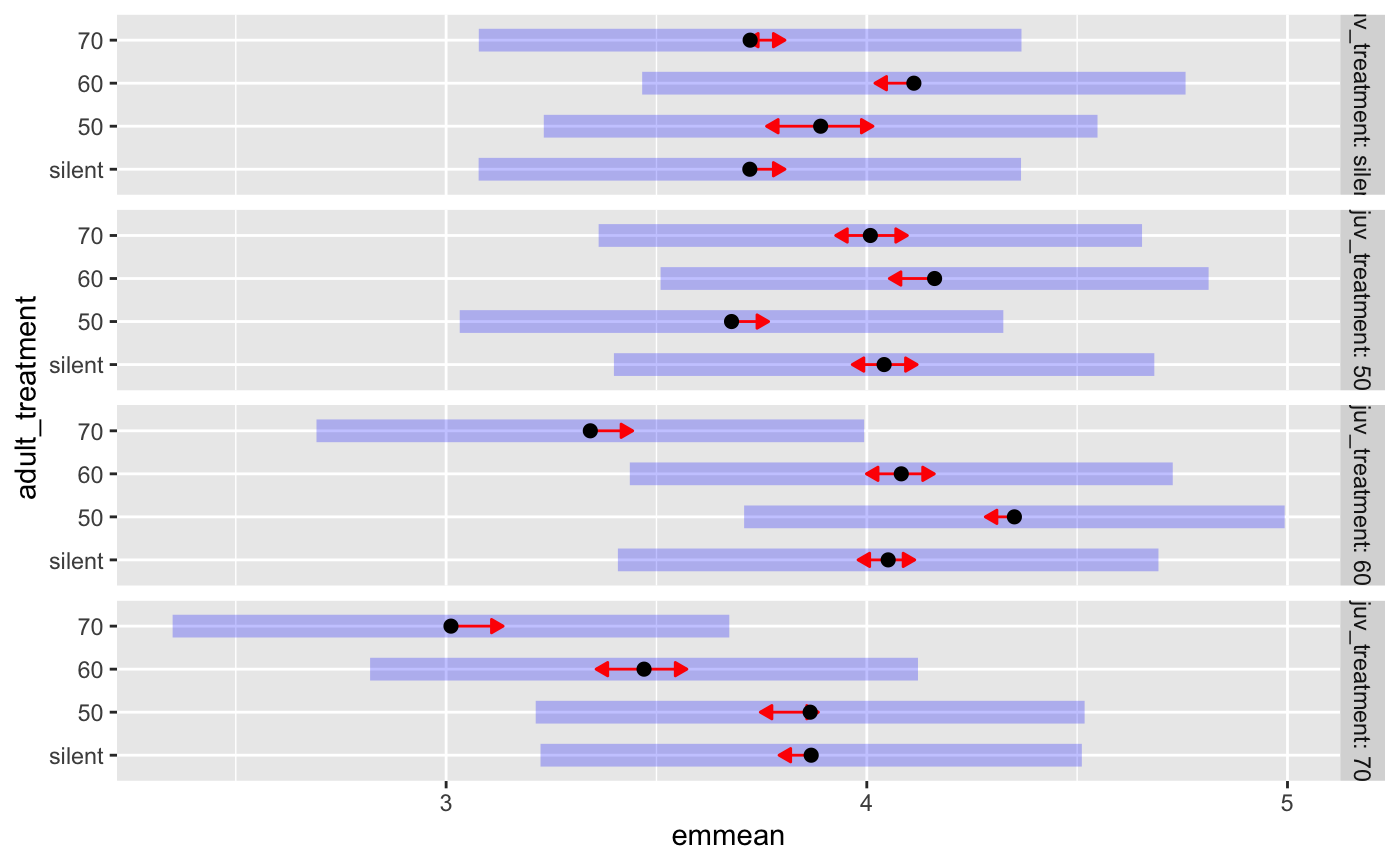


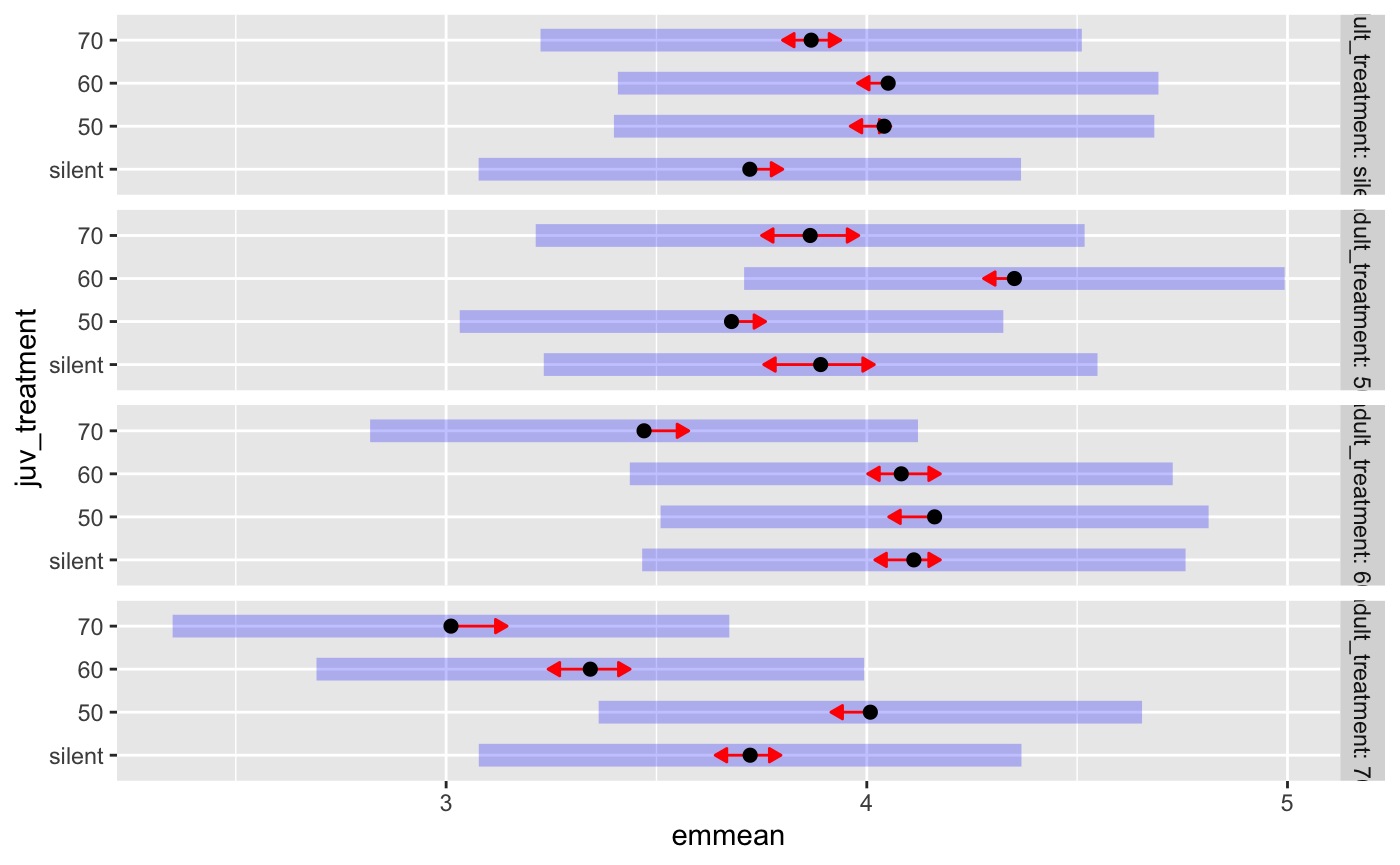


**Supplementary Figure 3.** Comparison arrow plots for the pairwise results of number of hatchlings. This is a graphic version of Supplementary Table 1 above. The top figure has the adult treatment for each category (horizontal facet) of juvenile treatment and the bottom figure has the juvenile treatment for each category of adult treatment. If the red arrows do not overlap within each category, then the number of hatchlings differs greatly between treatment type. If the red arrows do overlap, then there is no difference in the number of hatchlings. See more information this this vignette: https://cran.r-project.org/web/packages/emmeans/vignettes/xplanations.html#arrows


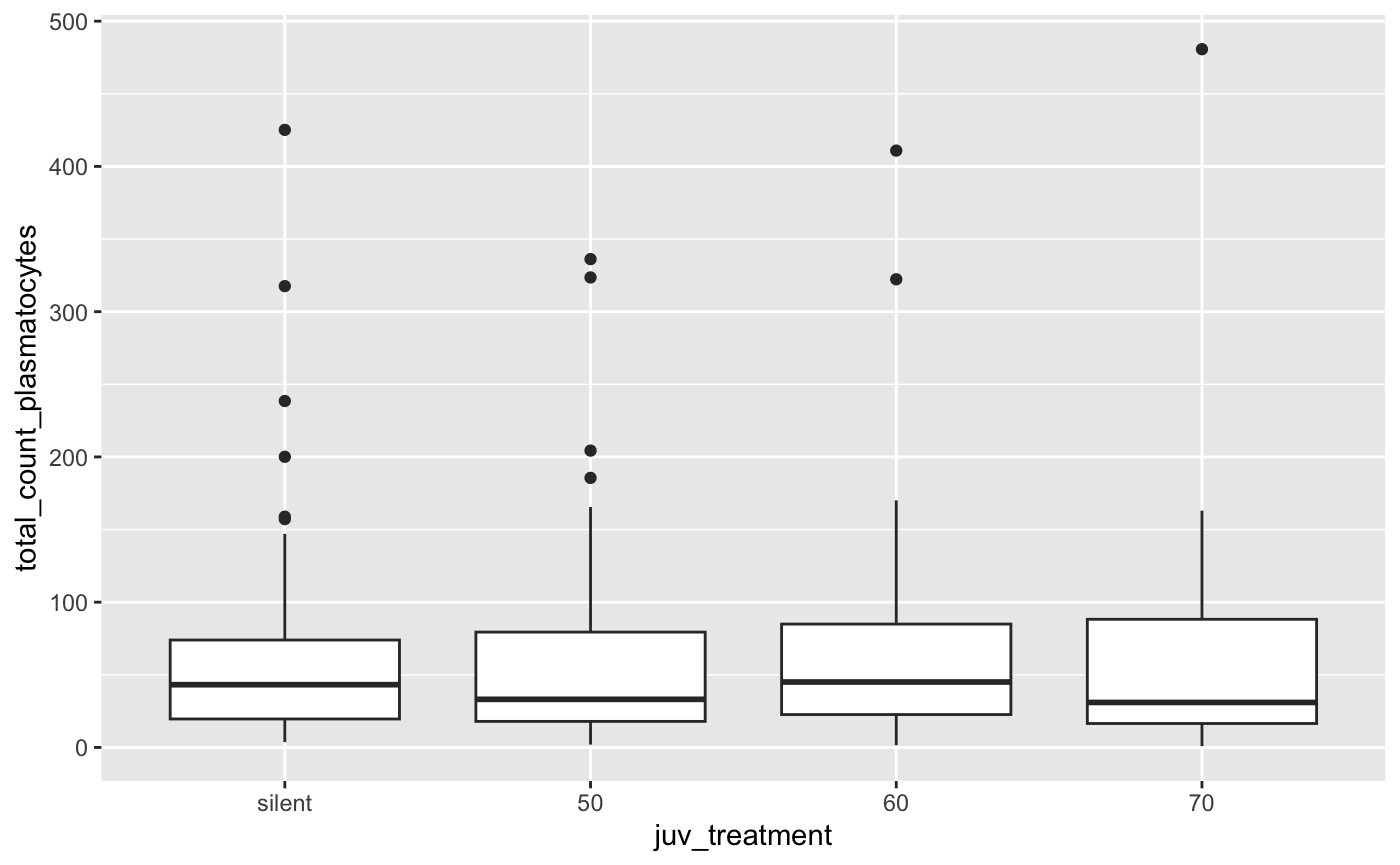


**Supplementary Figure 4.** The number of plasmatocytes was impacted by juvenile treatment (*X^2^* = 8.946, df = 3, p = 0.03) but the post hoc estimated marginal means test did not reveal any differences among juvenile treatments.

**Supplementary Table 2:** The amount of variance for each model that is attributed to among cohort differences.

| **Assay** | **Variance among cohorts** | **Standard deviation of the variance** |
| --- | --- | --- |
| Development time | 43.92 | 6.628 |
| Pronotum | 0.025 | 0.159 |
| Juvenile survival | 0.499 | 0.707 |
| Adult survival | 0.892 | 0.945 |
| Mated | 0.113 | 0.337 |
| Hatching success | 1.175 | 1.084 |
| Number of hatchlings | 0.517 | 0.719 |
| Sperm viability | 1.687 | 1.299 |
| Ovary mass | 0.0003 | 0.017 |
| Testes mass | 4.986 * 10^-07^ | 0.0007 |
| Accessory gland mass | 3.180 * 10^-06^ | 0.002 |
| Spermatophore mold mass | 8.402 x 10^-08^ | 0.0003 |
| Granulocytes | 0.073 | 0.271 |
| Plasmatocytes | 0.064 | 0.253 |
| Melanization | 0.002 | 0.041 |

**
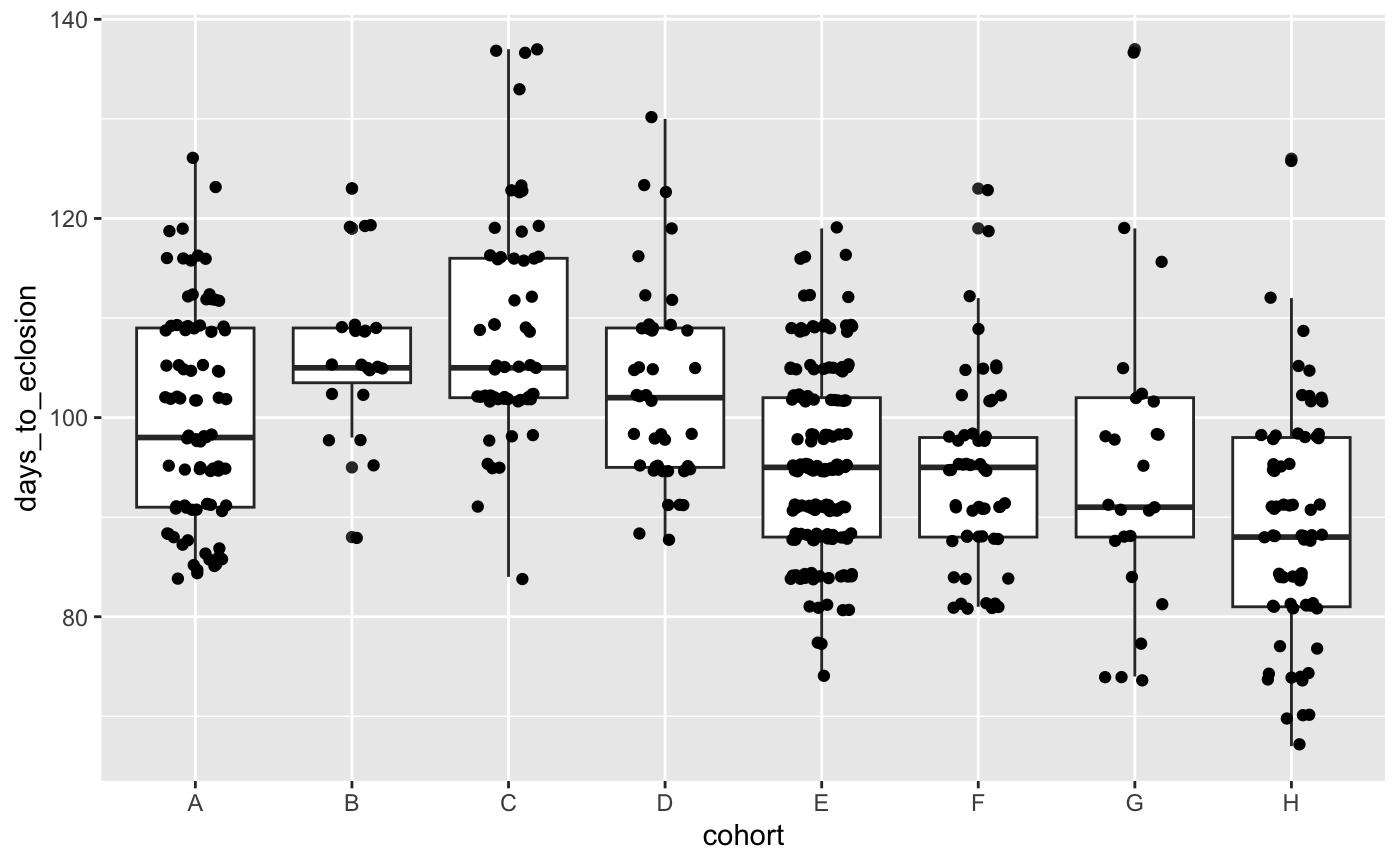
**

**Supplementary Figure 5.** There was substantial variance among cohorts in the development time model (see Supplementary Table 2), so here we plot variation in development time (days to eclosion) among cohorts (A-H). We think this is not particularly surprising and likely because of the way we measured development time. To calculate development time, we tracked the number of days from when the crickets entered the noise treatment to when they completed their final molt to adulthood (eclosion). Each cohort consisted of up to 420 crickets from the laboratory colony that were up to ~14 days old (the earliest we could transfer the fragile early instars). Our rearing methods in the lab are such that we knew that none of the crickets were older than 14 days, but some could have been younger. The age at which the animals were placed in treatments differed somewhat between cohorts, especially as there are natural pulses in when eggs lay and hatch.

**Supplementary Table 3.** Because there was substantial variance among cohorts (A-H) in development time, we ran a model identical to the development time model in the main text except that we included cohort as a fixed, rather than random effect. The model revealed that development time was strongly influenced by cohort (*X^2^* = 190.753, df = 7, p < 0.0001). Here we provide the full output of pairwise comparisons for the effect of cohort on development time to identify potential “outlier” cohort(s). We conclude that there is not one specific “outlier” cohort, given that numerous variable contrasts in the table below are significantly different

| contrast | estimate | SE | df | t.ratio | p.value |
| --- | --- | --- | --- | --- | --- |
| A - B | -4.89 | 2.27 | 506 | -2.158 | 0.3795 |
| A - C | -7.86 | 1.64 | 506 | -4.801 | 0.0001 |
| A - D | -2.94 | 1.8 | 506 | -1.631 | 0.7315 |
| A - E | 5.15 | 1.29 | 506 | 3.987 | 0.0019 |
| A - F | 7.13 | 1.66 | 506 | 4.296 | 0.0005 |
| A - G | 6.76 | 2.2 | 506 | 3.077 | 0.0454 |
| A - H | 11.46 | 1.57 | 506 | 7.305 | <.0001 |
| B - C | -2.98 | 2.37 | 506 | -1.257 | 0.914 |
| B - D | 1.95 | 2.5 | 506 | 0.78 | 0.9941 |
| B - E | 10.04 | 2.15 | 506 | 4.665 | 0.0001 |
| B - F | 12.02 | 2.38 | 506 | 5.05 | <.0001 |
| B - G | 11.65 | 2.79 | 506 | 4.177 | 0.0009 |
| B - H | 16.34 | 2.32 | 506 | 7.036 | <.0001 |
| C - D | 4.93 | 1.94 | 506 | 2.539 | 0.1815 |
| C - E | 13.02 | 1.48 | 506 | 8.795 | <.0001 |
| C - F | 14.99 | 1.8 | 506 | 8.351 | <.0001 |
| C - G | 14.62 | 2.31 | 506 | 6.324 | <.0001 |
| C - H | 19.32 | 1.71 | 506 | 11.309 | <.0001 |
| D - E | 8.09 | 1.66 | 506 | 4.882 | <.0001 |
| D - F | 10.07 | 1.96 | 506 | 5.128 | <.0001 |
| D - G | 9.7 | 2.43 | 506 | 3.998 | 0.0019 |
| D - H | 14.39 | 1.88 | 506 | 7.641 | <.0001 |
| E - F | 1.97 | 1.5 | 506 | 1.313 | 0.8939 |
| E - G | 1.6 | 2.08 | 506 | 0.773 | 0.9944 |
| E - H | 6.3 | 1.4 | 506 | 4.487 | 0.0002 |
| F - G | -0.37 | 2.32 | 506 | -0.159 | 1 |
| F - H | 4.33 | 1.74 | 506 | 2.489 | 0.2023 |
| G - H | 4.7 | 2.28 | 506 | 2.064 | 0.4398 |

**
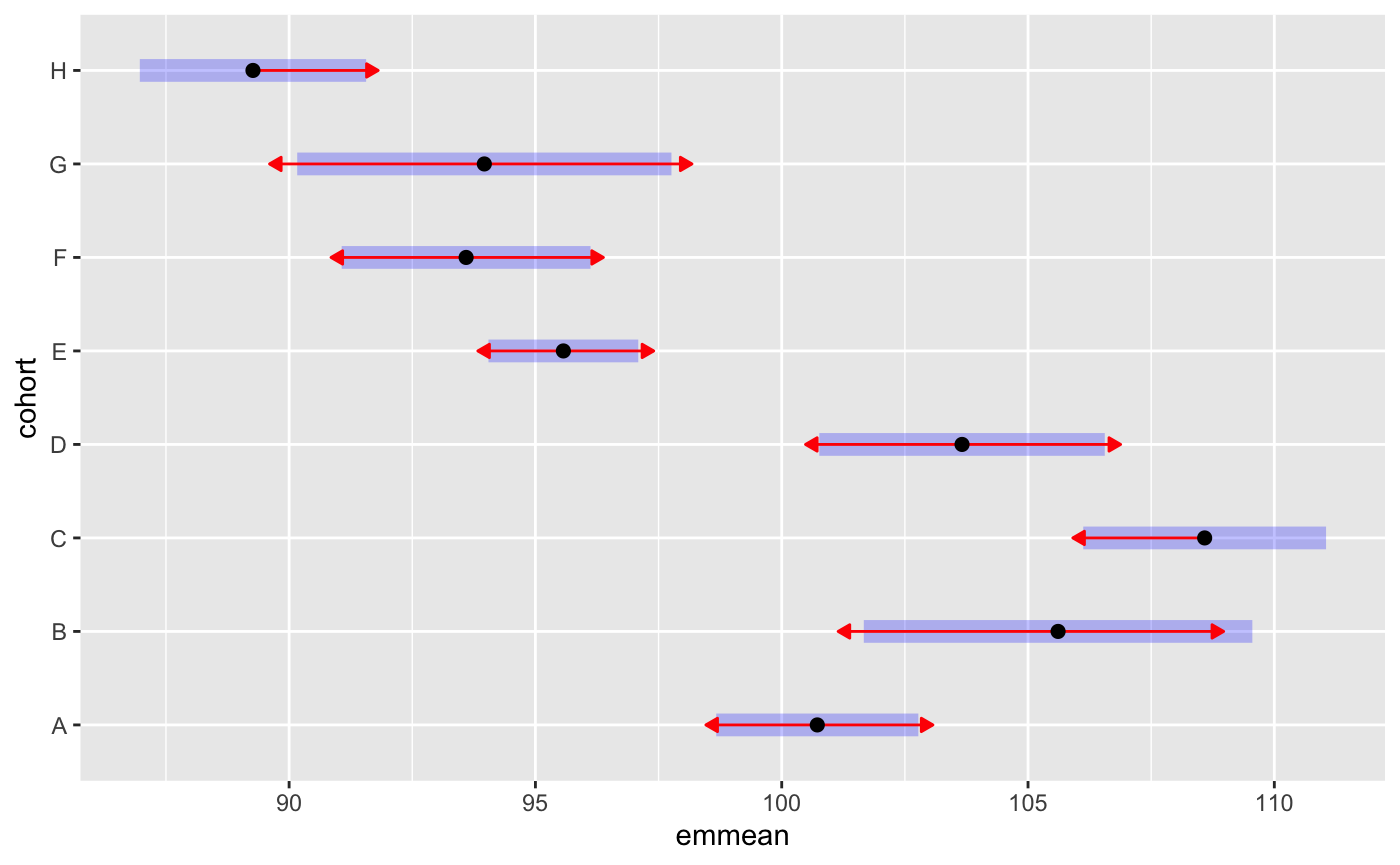
**

**Supplementary Figure 6.** Comparison arrow plots for the pairwise results of the effect of cohort on development time. This is a graphic version of Supplementary Table 3 above. If the red arrows do not overlap within each category, then there is a strong difference between cohorts. See more information this this vignette: https://cran.r-project.org/web/packages/emmeans/vignettes/xplanations.html#arrows

###


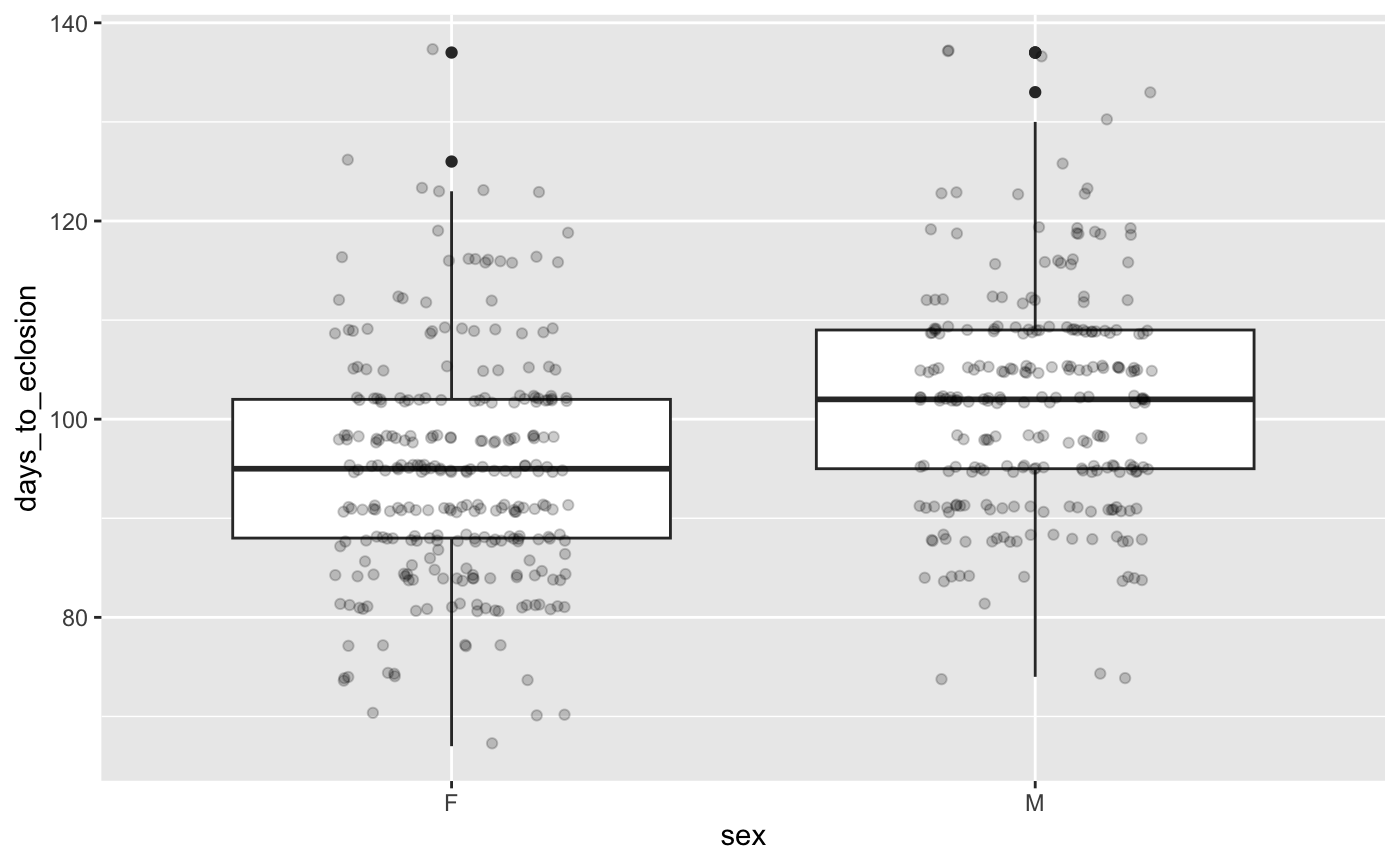


**Supplementary Figure 7.** Females developed to adulthood more quickly than males (avg 94.3 vs 101.6 days; *X^2^* = 74.403, df = 1, p < 0.0001).


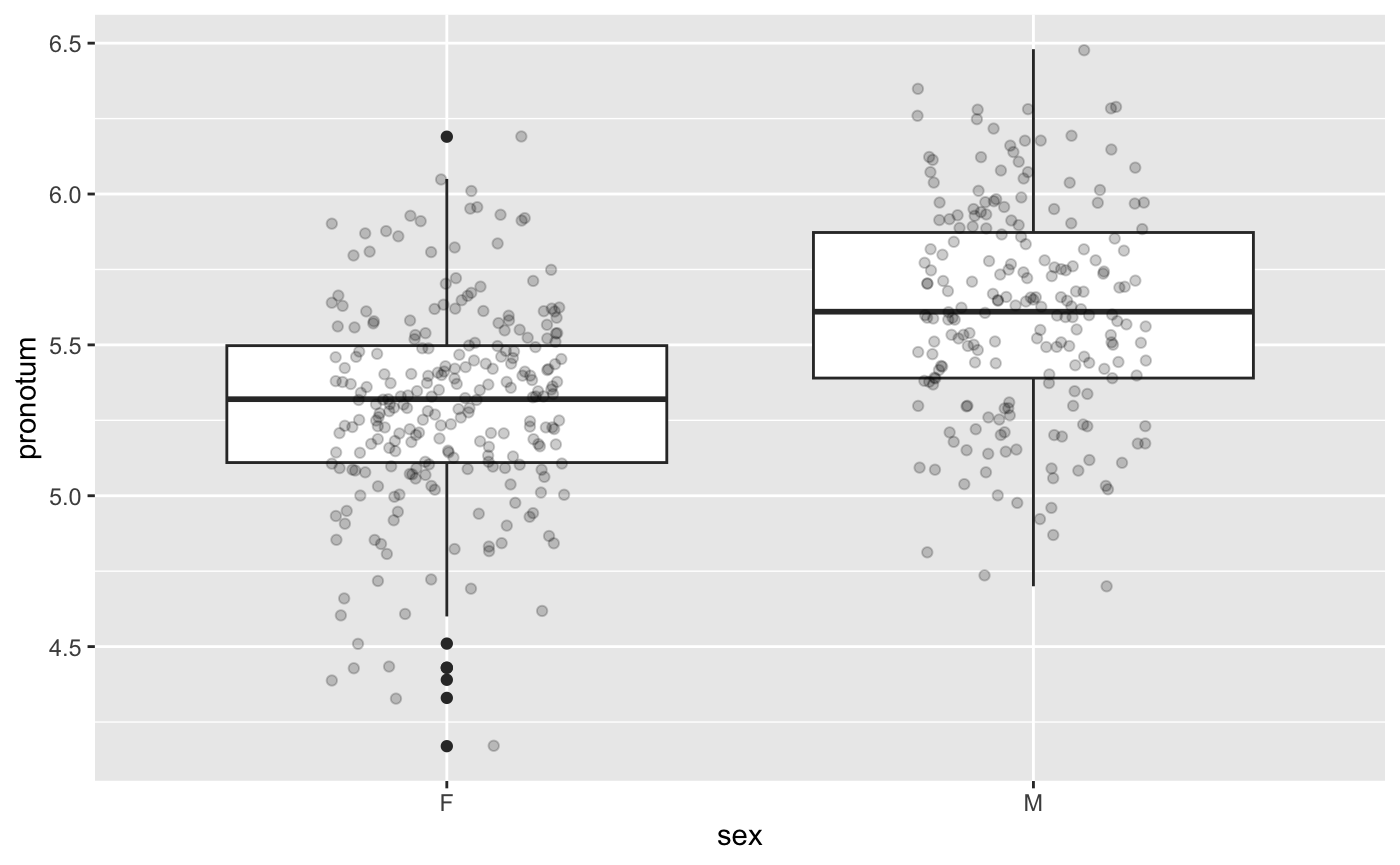


**Supplementary Figure 8.** Females were smaller than males (avg 5.303 vs 5.612mm; *X^2^* = 105.097, df = 1, p < 0.0001).

**
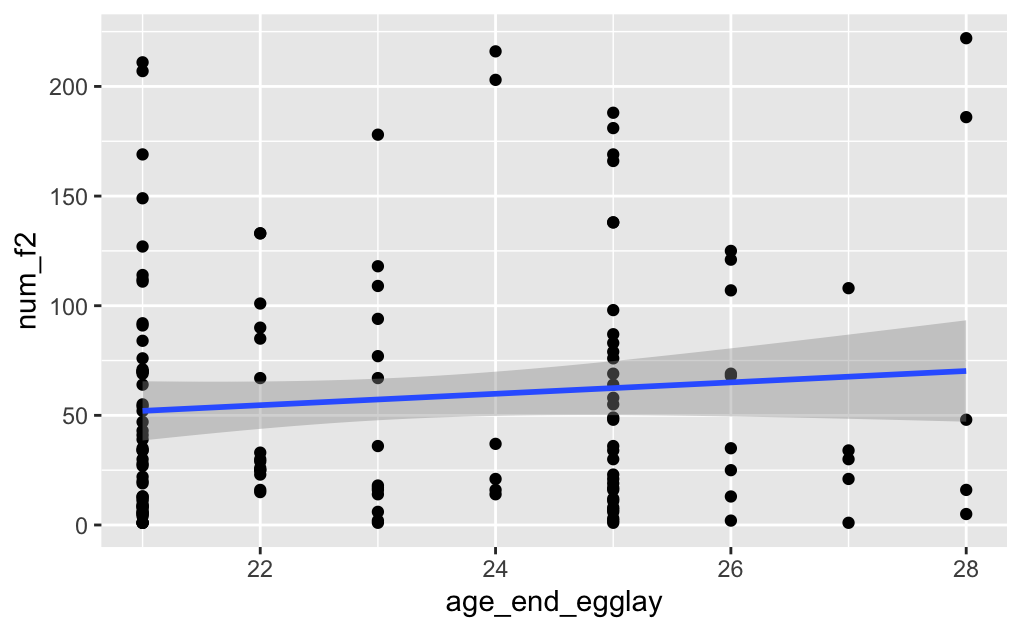
**

**Supplementary Figure 9.** Of females that had at least one offspring, older ones had more offspring than younger ones (*X^2^* = 34.118, df = 1, p < 0.0001).


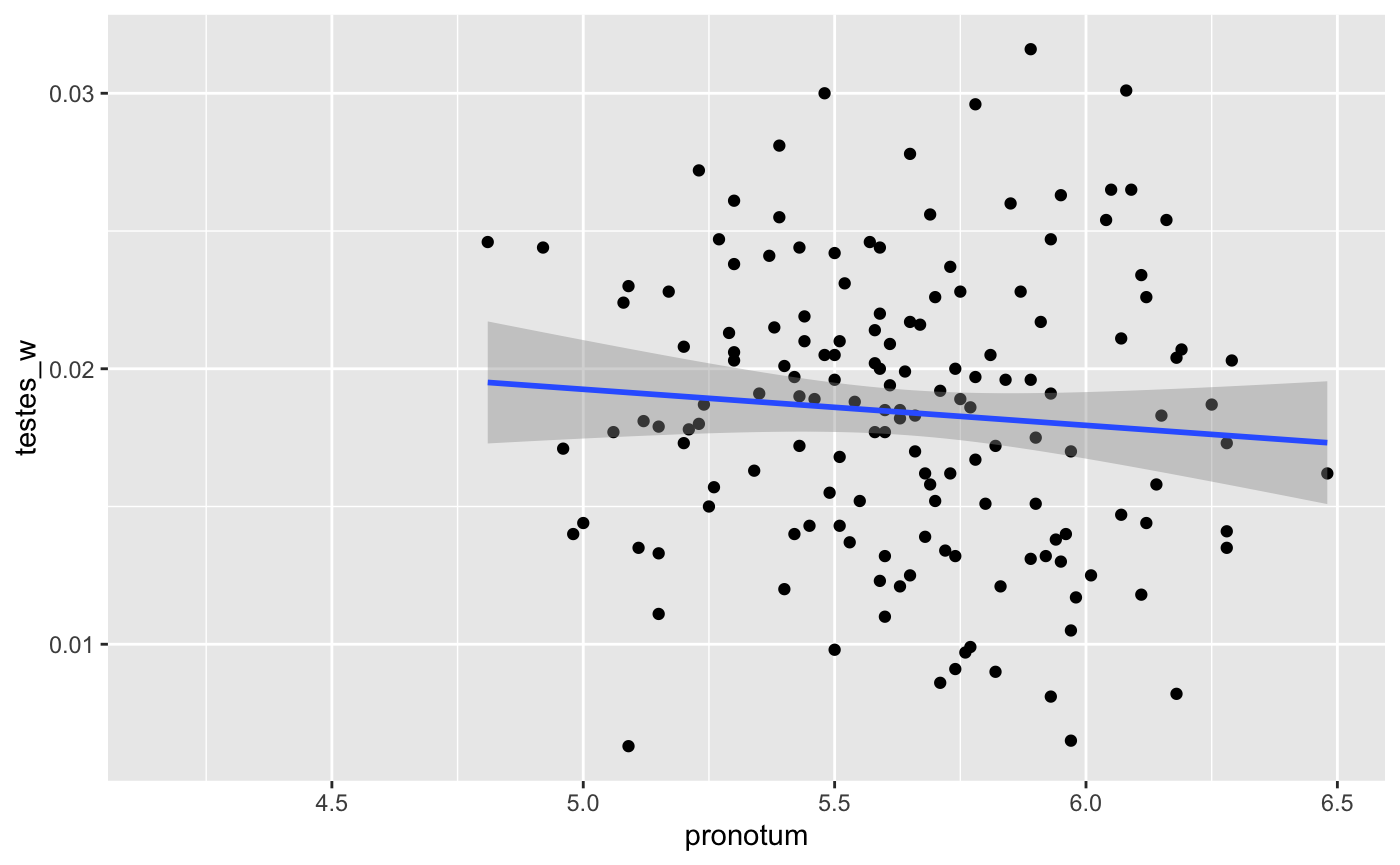


**Supplementary Figure 10.** Larger males had smaller testes (*X^2^* = 12.541, df = 1, p = 0.0004).

**
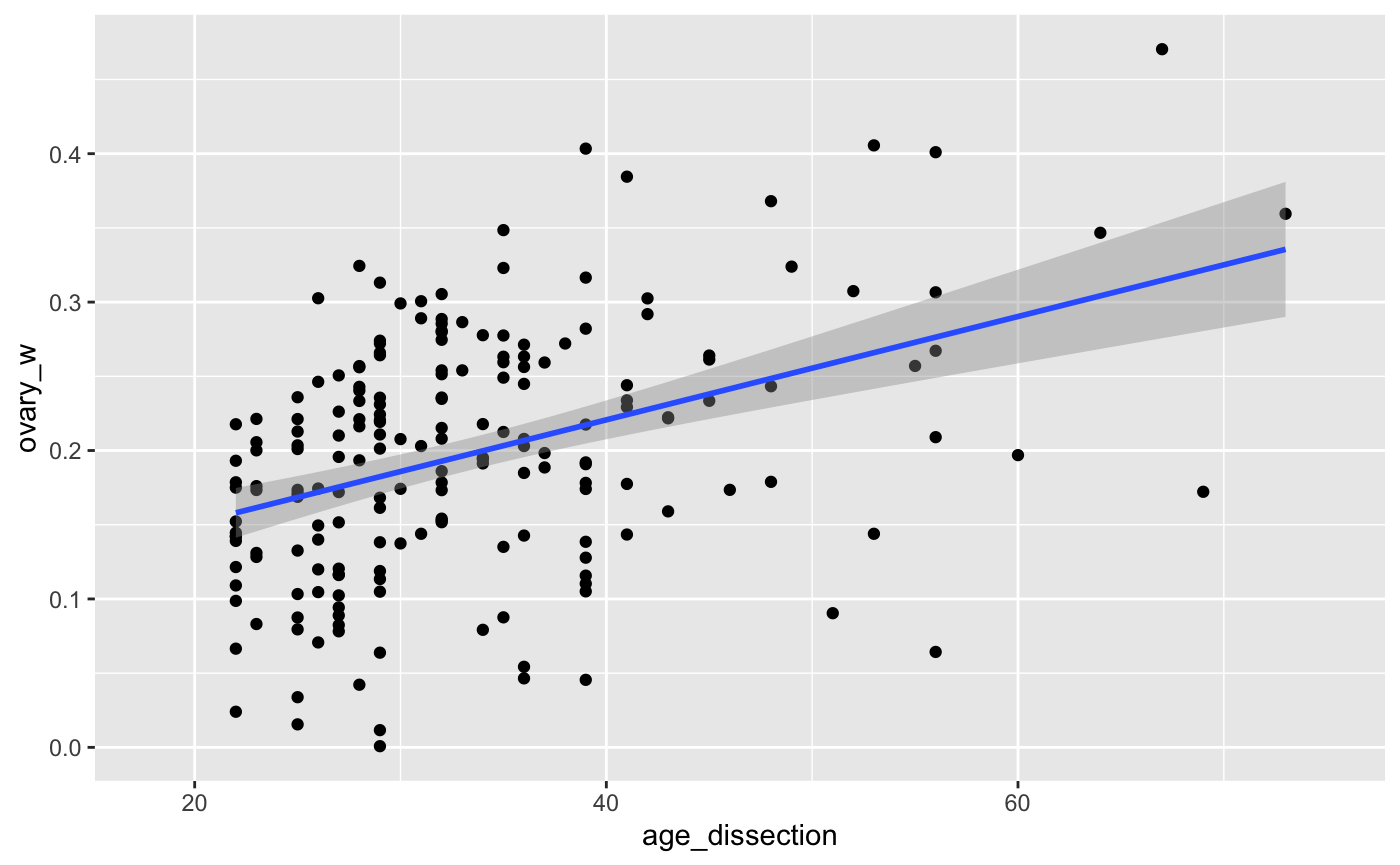
**

**Supplementary Figure 11.** Older crickets had larger ovaries (*X^2^* = 13.94, df = 1, p = 0.0002).


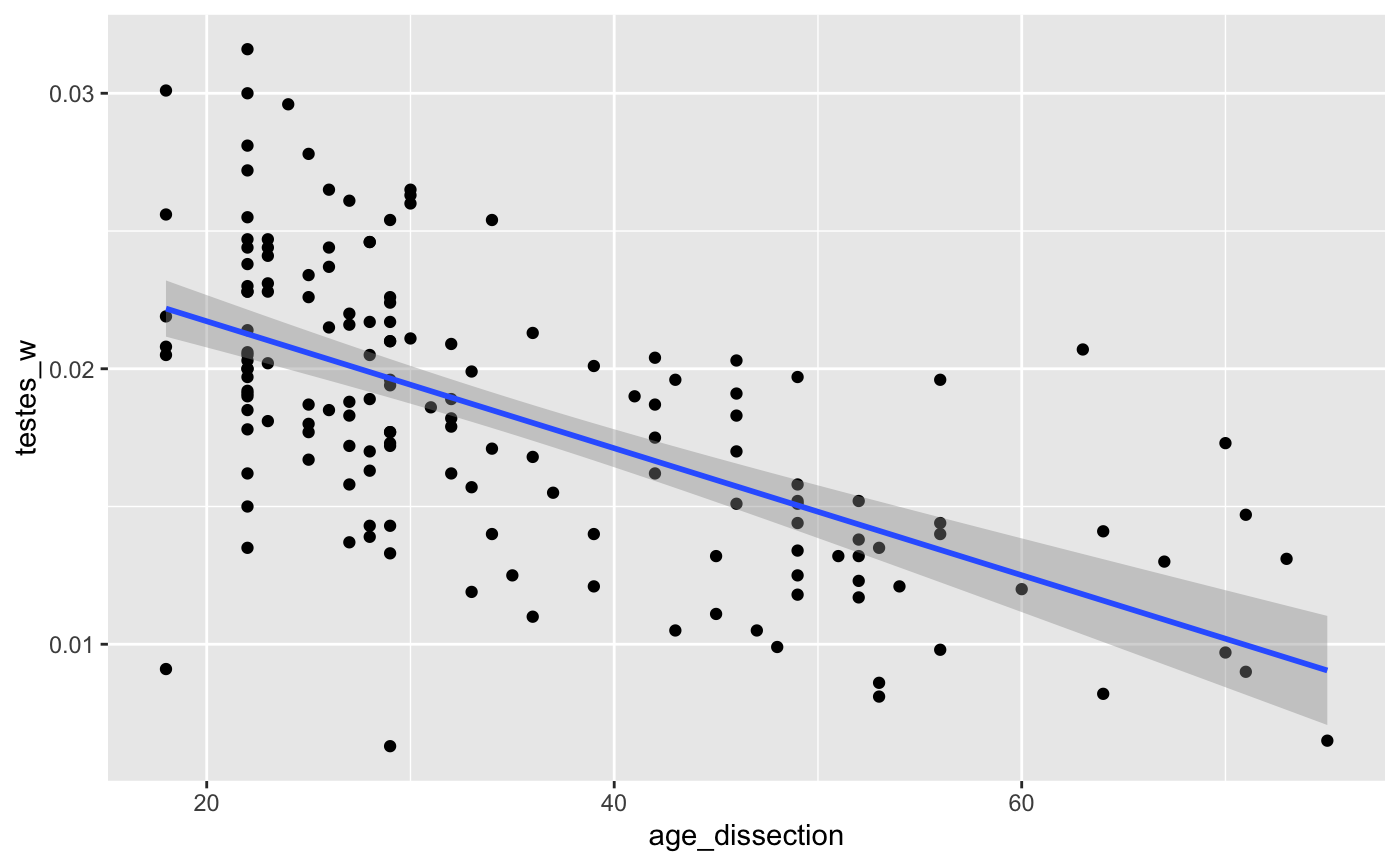


**Supplementary Figure 12.** Older males had smaller testes (*X^2^* = 79.974, df = 1, p < 0.0001).
